# Palmitoylation of Gephyrin Controls Receptor Clustering and Plasticity of GABAergic Synapses

**DOI:** 10.1371/journal.pbio.1001908

**Published:** 2014-07-15

**Authors:** Borislav Dejanovic, Marcus Semtner, Silvia Ebert, Tobias Lamkemeyer, Franziska Neuser, Bernhard Lüscher, Jochen C. Meier, Guenter Schwarz

**Affiliations:** 1 Institute of Biochemistry, Department of Chemistry, University of Cologne, Cologne, Germany; 2 RNA Editing and Hyperexcitability Disorders Helmholtz Group, Max Delbrück Center for Molecular Medicine, Berlin, Germany; 3 Cologne Excellence Cluster on Cellular Stress Responses in Aging-Associated Diseases (CECAD), University of Cologne, Cologne, Germany; 4 Department of Biology and Department of Biochemistry and Molecular Biology, Pennsylvania State University, University Park, Pennsylvania, United States of America; 5 Center for Molecular Medicine Cologne (CMMC), University of Cologne, Cologne, Germany; Thomas Jefferson University, United States of America

## Abstract

Postsynaptic scaffolding proteins regulate coordinated neurotransmission by anchoring and clustering receptors and adhesion molecules. Gephyrin is the major instructive molecule at inhibitory synapses, where it clusters glycine as well as major subsets of GABA type A receptors (GABA_A_Rs). Here, we identified palmitoylation of gephyrin as an important mechanism of strengthening GABAergic synaptic transmission, which is regulated by GABA_A_R activity. We mapped palmitoylation to Cys212 and Cys284, which are critical for both association of gephyrin with the postsynaptic membrane and gephyrin clustering. We identified DHHC-12 as the principal palmitoyl acyltransferase that palmitoylates gephyrin. Furthermore, gephyrin pamitoylation potentiated GABAergic synaptic transmission, as evidenced by an increased amplitude of miniature inhibitory postsynaptic currents. Consistently, inhibiting gephyrin palmitoylation either pharmacologically or by expression of palmitoylation-deficient gephyrin reduced the gephyrin cluster size. In aggregate, our study reveals that palmitoylation of gephyrin by DHHC-12 contributes to dynamic and functional modulation of GABAergic synapses.

## Abstract 2

Gephyrin, the principal scaffolding protein at inhibitory synapses, needs to be palmitoylated in order to cluster and to assemble functional synapses.

## Author Summary

Efficient signal transmission at synapses is essential for higher brain functions. Inhibitory signaling in the brain takes place primarily at GABA (γ-aminobutyric acid)-ergic synapses. GABA type A receptors (GABA_A_Rs) are clustered at the postsynaptic side by a scaffold composed of the peripheral membrane protein gephyrin. We demonstrate that gephyrin is modulated by palmitoylation, a reversible posttranslational fatty acid modification. Palmitoylation facilitates the membrane association of gephyrin and is therefore essential for normal clustering of gephyrin at GABAergic synapses. Reciprocally, palmitoylation of gephyrin is regulated by GABA_A_R activity. Of the 23 known palmitoyl transferases that catalyze the palmitoylation of proteins in human cells, we identified one enzyme, DHHC-12, to specifically modify gephyrin. Our results provide a new aspect to the posttranslational control of synaptic plasticity.

## Introduction

Regulated signal transmission in the central nervous system is essential for higher brain function [1],[2]. Inhibitory signaling in the brain primarily takes place at glycinergic and GABA (γ-aminobutyric acid)-ergic synapses. Glycine receptors and a subset of GABA type A receptors (GABA_A_Rs) are clustered at the synapse by a scaffold of the peripheral membrane protein gephyrin as a major postsynaptic component [3]. Loss of gephyrin leads to the loss of postsynaptic glycine and GABA_A_R clustering [4]–[6].

Dynamic regulation of the number of GABA_A_Rs at synapses provides a key mechanism for functional plasticity of inhibitory synapses [7]. Recently, it was shown that gephyrin exerts a major influence on experience-dependent plasticity of inhibitory synapses *in vivo* [8],[9]. Gephyrin phosphorylation has been demonstrated to contribute to functional regulation of GABAergic synapses [10],[11]. Phosphorylation of serine residues 268 and 270 modulate the density and size of gephyrin clusters in hippocampal neurons and hence contribute to synaptic plasticity. However, little is known about the mechanisms controlling the association of gephyrin with the postsynaptic membrane.

Lipid modification of proteins serves as a major mechanism controlling cellular localization and association of proteins with membranes [12]. In particular, reversible palmitoylation has emerged as the most frequent lipid modification of synaptic proteins, with diverse effects on protein trafficking, neuronal development, and synaptic plasticity [13]. Palmitoylation has most extensively been studied in the context of proteins that function at excitatory synapses. Among these, reversible palmitoylation of the scaffolding protein PSD-95 has been implicated in activity-dependent plasticity of excitatory synapses [14],[15]. In mammalian cells, palmitoylation is catalyzed by a family of 23 palmitoyl transferases that share a conserved DHHC (Asp-His-His-Cys) motif [16]. One of these, DHHC-3/GODZ, has been identified as a palmitoyl transferase of the γ2 subunit of GABA_A_Rs that regulates the formation and function of GABAergic synapse [17],[18]. However, little is known about the role of other palmitoylated substrates at inhibitory synapses.

A recent proteomic approach identified gephyrin as a potential palmitoylated substrate at inhibitory synapses [19]. Here, we confirm and extend this finding. In particular we show that gephyrin is palmitoylated *in vivo*, which determines whether gephyrin is membrane bound or soluble and thus regulates the size of postsynaptic gephyrin clusters. Functional studies in neurons identified DHHC-12 as the primary palmitoyl transferase. Palmitoylation levels are regulated by GABA_A_R activity. Our results identify palmitoylation of gephyrin as a key mechanism underlying functional plasticity of GABAergic synapses.

## Results

### Gephyrin Is Palmitoylated *in Vivo*

**Figure 1:**
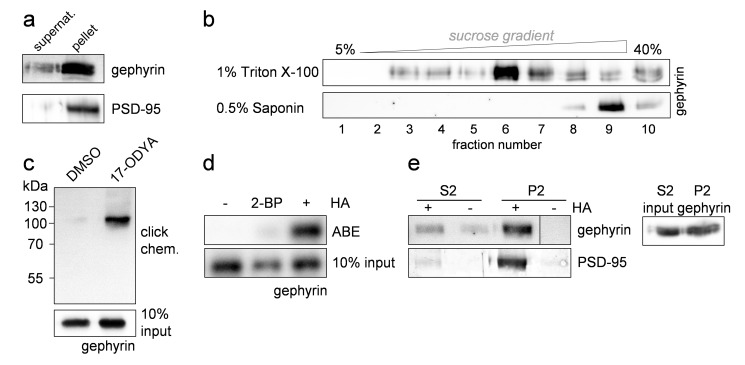
Gephyrin is palmitoylated *in vivo*. (a) Mouse brain lysates were fractionated via ultracentrifugation and immunoblotted with gephyrin and PSD-95–specific antibodies. (b) Representative immunoblots of enriched synaptosomes after extraction with Triton X-100 or Saponin, overlayed with a discontinuous sucrose gradient and ultracentrifugation. Ten fractions were taken from top to bottom. (c) Gephyrin palmitoylation detected by metabolic labeling with 17-ODYA followed by click chemistry and affinity purification. (d) Basal palmitoylation of gephyrin in primary hippocampal neurons demonstrated by the ABE assay. Replacing hydroxylamine (HA) with a Tris-buffer (−HA) served as an internal negative control to proof efficient blockade of thiols. Palmitoylation of gephyrin was blocked with 50 µM 2-BP for 14 h. (e) Gephyrin palmitoylation status in the cytosolic (S2) compared to synaptosomal (P2) fraction analyzed by the ABE assay. Lanes of the top panel have been spliced from the same membrane. Volumes of the S2/P2 fractions have been adjusted to similar amounts of gephyrin (right panel). Experiment was performed with two individual synaptosomal preparations with essentially the same results.

Gephyrin anchors major subsets of GABA_A_Rs in the postsynaptic membrane [3]. We performed ultracentrifugation-based subcellular fractionation of mouse brain lysates and found gephyrin mainly in the pellet fraction, demonstrating that it is mostly associated with membranes (Figure 1a). Furthermore, gephyrin was able to “float” in Triton X-100 solubilized mouse brain synaptosomes (Figure 1b). Detergent-resistant membranes (DRMs) mainly consist of cholesterol [20]. Upon solubilization of DRMs with the cholesterol-complexing agent saponin, gephyrin no longer floated in sucrose gradients, indicating that it is likely lipid-modified.

Protein palmitoylation is implicated in targeting proteins to DRMs [21]. Metabolic labeling of gephyrin-expressing Sf9 cells with the palmitate analogue 17-octadecynoic acid (17-ODYA) followed by the application of click-chemistry and affinity-purification revealed prominent gephyrin palmitoylation (Figure 1c). In addition, native neuronal gephyrin was found to be palmitoylated, as shown by acyl-biotin exchange (ABE) assays of cultured hippocampal neurons (Figure 1d). Treatment of neurons with 50 µM of the DHHC enzyme blocker 2-bromopalmitate (2-BP) significantly reduced the palmitoylation level of gephyrin (Figure 1d). To assess the subcellular localization of palmitoylated gephyrin, we fractionated crude brain homogenates into synaptosome-enriched and cytosolic fractions, adjusted the volume of samples to process similar amounts of gephyrin, and performed ABE assays (Figure 1e). Similar to PSD-95, gephyrin was found to be palmitoylated mainly in the synaptosomal fraction, indicating that it is lipid-modified at synapses.

### Palmitoylation of Gephyrin Is Crucial for Normal Postsynaptic Clustering

**Figure 2:**
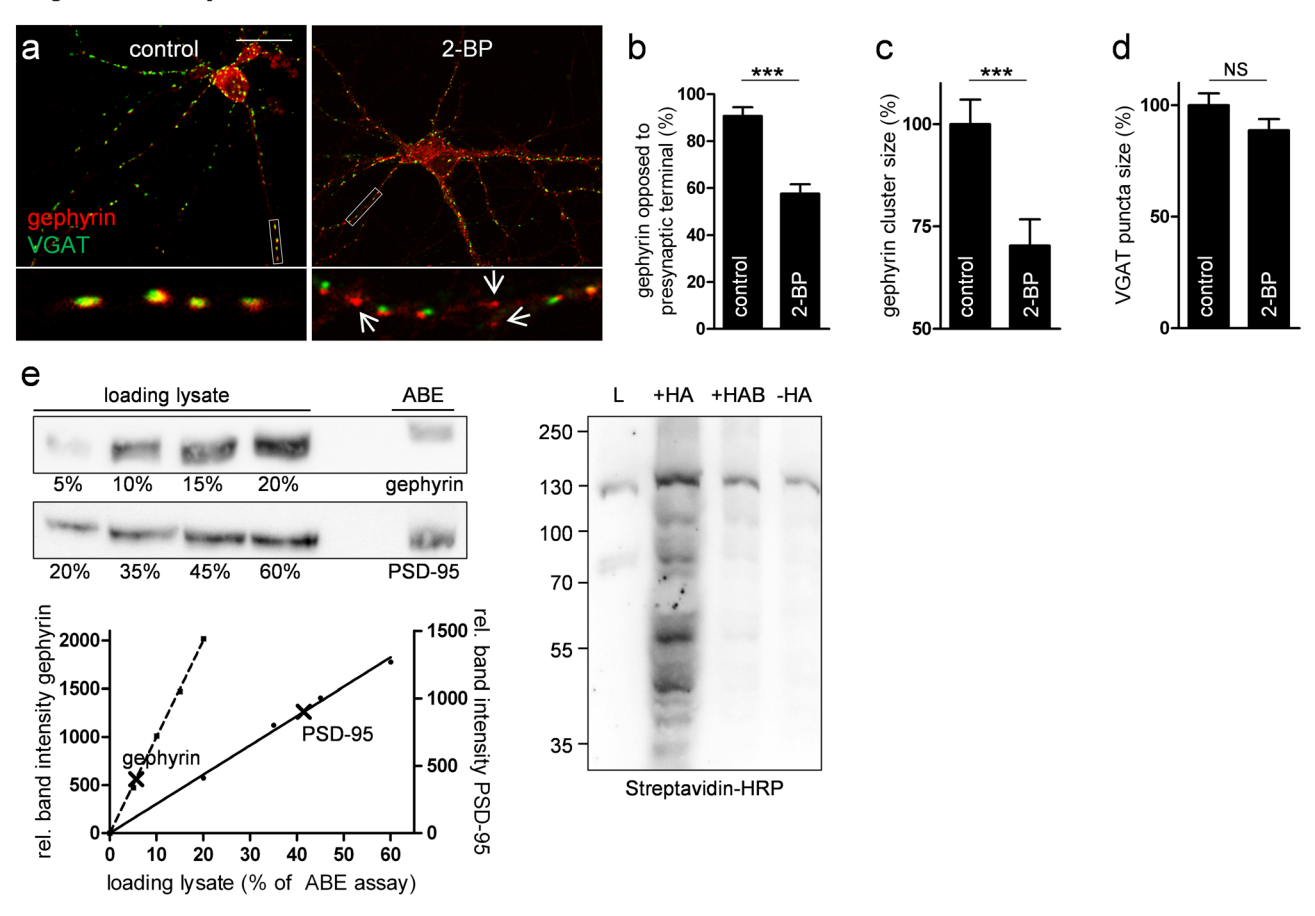
Palmitoylation of gephyrin is essential for clustering at GABAergic synapses. (a) Immunostaining of gephyrin (red) and VGAT (green) in primary hippocampal neurons. Insets are taken from 20 µm dendritic segments. Arrows point to nonsynaptic gephyrin clusters upon incubation with 2-BP. Scale bar, 20 µm. (b–d) Histograms showing quantitative comparisons of (b) gephyrin colocalization with VGAT, (c) gephyrin cluster size, and (d) VGAT puncta area in control and 2-BP–incubated cultures. (e) Quantification of percentage of palmitoylated proteins in mouse brains. Immunoblots with increasing volumes of ABE-assay–processed protein lysates were used to create a band-intensity standard curve for ABE-assay eluates of gephyrin and PSD-95. The experiment has been performed with three brains. (f′) Streptavidin-HRP immunoblot served as a specificity and accuracy control of the assay. L, lysate; +HA, samples processed with hydroxylamine; +HAB, +HA supernatant after Neutravidin beads sedimentation to prove efficient affinity-purification of biotinoylated proteins; −HA, internal control of the ABE assay without hydroxylamine.

We next investigated the role of gephyrin palmitoylation with respect to its localization and clustering at inhibitory synapses. Co-immunostaining of hippocampal neurons at 14 DIV indicated co-localization of gephyrin cluster immunoreactivity with punctate immunoreactivity for the presynaptic terminal marker vesicular, GABA transporter (VGAT, Figure 2a). In control neurons, gephyrin clusters were extensively colocalized with VGAT, whereas upon inhibition of palmitoylation with 2-BP, we frequently observed nonsynaptic gephyrin clusters (control, 90.71%±3.73%; 2-BP, 57.61%±4.0% gephyrin co-localization with VGAT; *n* = 3 cultures; *p*<0.001; Figure 2a,b). Although the size of gephyrin clusters was significantly reduced (70.35%±6.32% of control gephyrin; *n* = 3 cultures; *p*<0.001), 2-BP had no effect on the size of VGAT puncta (Figure 2c,d). In line with these results, cell surface biotinylation of 2-BP–treated neurons revealed significantly decreased surface expression of GABA_A_R subunits representative of synaptic receptors (α1, β2/3, and γ2), whereas the α5 subunit, representative of mainly extrasynaptic receptors that do not associate with gephyrin, was unaffected (Figure S1). Gephyrin complexes in homogenates from control and 2-BP–incubated neurons remained around ∼400–700 kDa in size, as demonstrated by size exclusion chromatography (Figure S2 and Text S1), suggesting that palmitoylation does not affect gephyrin oligomerization and thus does not explain the decrease in gephyrin cluster size upon inhibition of palmitoylation.

As gephyrin oligomers, which we previously characterized as hexamers and nonamers, seem to function as stable and individual units [22], we wondered how much gephyrin is palmitoylated in neurons. Therefore, increasing amounts of lysate were taken before affinity purification of ABE-processed brain samples and quantitated along with affinity-purified bead eluates by Western blotting. Quantification of the band intensities revealed that 7.5%±0.5% of gephyrin was palmitoylated; by comparison, the proportion of palmitoylated PSD-95 that does not form oligomers (Figure S2) was significantly higher (Figure 2e).

### Palmitoylation of Gephyrin at Cys212 and Cys284 Is Essential for Clustering at GABAergic Synapses

**Figure 3:**
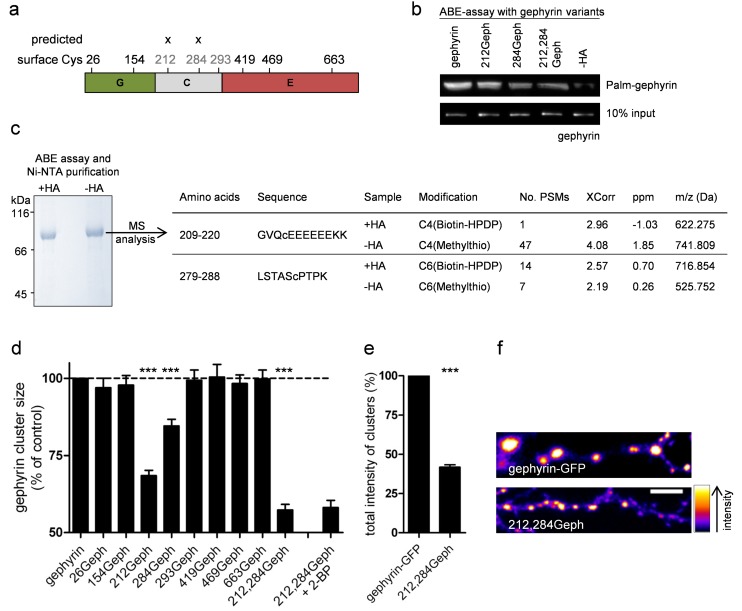
Palmitoylation on Cys212 and Cys284 is essential for gephyrin localization and clustering in neurons. (a) Scheme of gephyrin domain architecture with surface-exposed and Palm-CSS 3.0–predicted cysteine residues. (b) Immunoprecipitation of ABE-assay–processed gephyrin cysteine-to-serine mutants expressed in HEK293 cells. Streptavidin-HRP shows palmitoylation status of individual variants, whereas GFP-immunoblot was used as the loading control. Experiment was repeated three times with similar results. (c) SDS-PAGE of ABE-assay–processed and Ni-NTA affinity-purified gephyrin and LC-MS/MS analysis of the protein bands. (d) Quantitative comparison of cluster size of GFP-tagged WT and mutant gephyrin variants in primary hippocampal cultures. (e) Total intensity of WT gephyrin or 212,284Geph clusters (f) Representative dendrites of gephyrin-GFP and 212,284Geph. Scale bar, 5 µm. All quantifications are means ± SEM (***p<0.001 using Student's *t* test; NS, not significant). At least three independent cultures were used per experiment.

Gephyrin has a modular structure that is composed of an N-terminal G-, a central C-, and C-terminal E-domain [23]. To identify functionally relevant residues in gephyrin that are palmitoylated, we focused on surface-exposed cysteine residues on the G- and E-domain [24],[25] and the three Cys residues within the C-domain. This reduced the number of potentially palmitoylated residues from 12 to 8 (Figure 3a). Among these, only Cys212 and Cys284 in the C-domain were predicted as potentially palmitoylated by the CSS-Palm 3.0 program [26]. All eight Cys residues (Figure 3a) were replaced by serine, either individually or in combination, using GFP-tagged gephyrin-GC and full-length gephyrin constructs—hereafter termed “number of residue”Geph(-GC). Upon expression of the Cys variants in HEK293 cells and analyses of palmitoylation by ABE assays, we found reduced gephyrin palmitoylation for 212Geph and 284Geph and the strongest reduction in gephyrin palmitoylation in the Cys212/Cys284 double mutant variant (Figures 3b and S3). To confirm that Cys212 and 284 are palmitoylated, we performed mass spectrometry analysis of ABE-processed gephyrin expressed in Sf9 cells. We identified biotin moieties selectively on Cys212- and Cys284-containing peptides (Figure 3c).

Next, we compared the synaptic clustering properties of the eight GFP-tagged cysteine-to-serine gephyrin variants and a wild-type (WT) construct in transfected primary hippocampal neurons. Among the eight mutant variants, only 212Geph and 284Geph showed a significant decrease in gephyrin cluster sizes, and the effect of these mutations was additive in the 212,284Geph variant (212Geph, 68.45%±1.76%; 284Geph, 84.58%±2.14%; 212,284Geph, 57.34%±1.79% of gephyrin-GFP; *n* = 5 cultures; *p*<0.001). All other variants were indistinguishable from the WT gephyrin construct (Figure 3d). The total fluorescence of 212,284Geph clusters was significantly reduced to 41.8%±1.5%, suggesting 212,284Geph clusters contained less than half the number of gephyrin molecules compared to clusters formed by WT gephyrin (Figure 3e,f). The fact that the cluster size of 212,284Geph was not further decreased by 2-BP treatment (58.11%±2.34% of gephyrin-GFP; Figure 3d) suggests that (i) residues Cys212 and Cys284 are palmitoylated in neurons, (ii) clustering of transfected palmitoylation-deficient gephyrin remains unaffected by endogenous gephyrin, and (iii) gephyrin clustering is unaffected by palmitoylation-independent effects of 2-BP.

### Palmitoylation of Gephyrin Is Essential for Clustering at GABAergic Synapses

**Figure 4:**
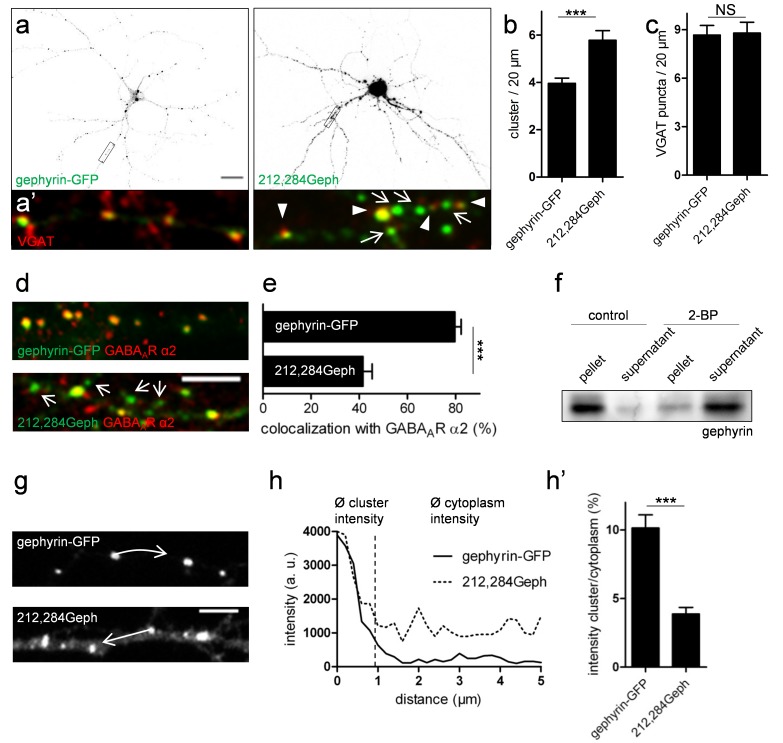
Morphological analysis of palmitoylation-deficient gephyrin variants in primary hippocampal neurons. (a) Representative examples are illustrated for WT gephyrin-GFP and 212,284Geph. Scale bar, 20 µm. (a′) Insets demonstrate postsynaptic clustering of gephyrin by colocalization with VGAT (red). 212,284Geph clusters show postsynaptic (arrowheads) and nonsynaptic (arrows) localization. (b, c) Quantitative analysis of (b) gephyrin cluster numbers and (c) VGAT puncta in gephyrin-GFP and 212,284Geph-transfected primary hippocampal neurons. (d) Colocalization of gephyrin-GFP and 212,284Geph clusters with GABA_A_R α2 immunofluorescence. Arrows show 212,284Geph clusters that are not localized at GABAergic synapses. Scale bar, 5 µm. (e) Quantitative analysis of cluster colocalized with α2-containing GABA_A_Rs. (f) Representative immunoblot of subcellular fractionation of 14 DIV cultured hippocampal neurons after incubation with 50 µM 2-BP or the solvent. (g–h) Quantitative line-scan analyses of fluorescence intensities ranging from individual clusters towards the cytosol as indicated by white arrows. Scale bar, 5 µm. (h′) Histogram shows quantification of cluster/cytoplasm intensity rations in gephyrin-GFP compared to 212,284Geph-transfected dendrites. All data are means ± SEM (***p<0.001 using Student's *t* test; NS, not significant). At least three independent cultures were used for the quantifications.

To assess the relevance of Cys212,284 palmitoylation for the accumulation of gephyrin at synapses, we expressed gephyrin-GFP and 212,284Geph in hippocampal neurons and immunostained for VGAT (Figure 4a,a′). Similar to effects of 2-BP treatment on endogenous gephyrin, transfected palmitoylation-deficient gephyrin accumulated in both synaptically and nonsynapticaly localized clusters (Figure 4a–c). In addition, 212,284Geph-expressing neurons showed a significantly increased density of clusters compared to gephyrin-GFP–transfected controls (gephyrin-GFP, 3.96%±0.22%; 212,284Geph, 5.77%±0.41% clusters/20 µm; *n* = 4 cultures; *p*<0.001; Figure 4a′,b), whereas the number of VGAT puncta was unchanged (Figure 4c). Importantly, supernumerary clusters of 212,284Geph were not colocalized with punctate immunoreactivity for α2-subunit–containing GABA_A_Rs (Figure 4d; gephyrin-GFP, 79.67%±2.62%; 212,284Geph, 41.49%±3.83% colocalization; *n* = 3 cultures; *p*<0.001; Figure 4e), suggesting that these gephyrin clusters were nonsynaptic and nonfunctional.

We hypothesized that mislocalized, nonsynaptic gephyrin clusters were not associated with the postsynaptic membrane and had instead accumulated in the soluble fraction. To test this idea, we fractionated 2-BP–treated and control primary neuron homogenates into soluble cytosolic and membrane-bound fractions. Compared to control cultures where gephyrin was mainly found in the insoluble pellet, 2-BP–incubated cultures revealed gephyrin enriched in the supernatant fraction (Figure 4f). Additionally, we quantified the ratio of clustered to cytoplasmic gephyrin in gephyrin-GFP and 212,284Geph-transfected neurons by line scan analyses of confocal micrographs. 212,284Geph showed strong fluorescence in the dendritic cytoplasm, which was not seen for gephyrin-GFP or endogenous gephyrin (Figure 4g,h, compare also to Figures 2a and 3f). Consistently, the cluster/cytoplasm intensity ratio of 212,284Geph compared to WT gephyrin was significantly decreased (gephyrin-GFP, 10.1%±0.9%; 212,284Geph, 3.9%±0.5%; *p*<0.001; *n* = 3 cultures; Figure 4h,h′). Thus, we conclude that palmitoylation of gephyrin is essential for postsynaptic cluster formation in neurons.

### DHHC-12 Is the Main Gephyrin-Palmitoylating Enzyme

**Figure 5:**
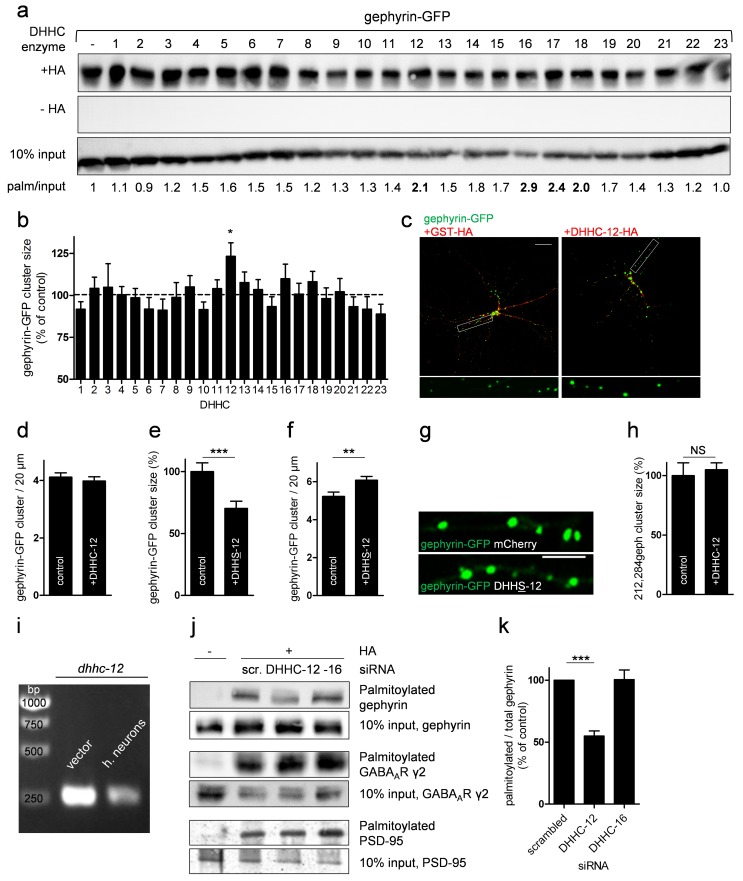
Gephyrin is palmitoylated by DHHC-12. (a) Individual HA-tagged DHHC proteins or GST-HA as control were co-expressed with gephyrin-GFP for 24 h in HEK293 cells and analyzed with the ABE assay. Omitting hydroxylamine demonstrated specify of the assay. (b) Gephyrin-GFP was co-expressed with individual HA-tagged DHHC enzymes or GST-HA as control in primary hippocampal neurons, and gephyrin cluster size was quantified by confocal laser scanning microscopy. In the histogram, DHHC enzymes are labeled according to [16]. (c) Representative images show phenotypical differences of gephyrin-GFP clusters in DHHC-12–expressing neurons compared to GST-expressing controls. Scale bar, 10 µm. (d) Gephyrin-GFP cluster density after 24 h expression of DHHC-12 compared to control neurons. (e–g) Quantification of gephyrin-GFP cluster size and density and representative image upon expression of dominant-negative DHHS-12–HA compared to mCherry-expressing control neurons. Scale bar, 5 µm. (h) Quantitative analysis of 212,284Geph cluster size in control mCherry and DHHC-12-HA–expressing neurons. (i) Expression of *dhhc12* mRNA in cultured hippocampal neurons (h. neurons) was validated by PCR using cDNA prepared from neurons. A plasmid encoding the respective *dhhc* gene was used as control. (j) Primary hippocampal neurons were grown in medium containing cell-penetrating siRNAs to knock down expression of DHHC-12 or DHHC-16. Lysates were subjected to ABE, and the palmitoylation level of gephyrin, GABA_A_R γ2, and PSD-95 was analyzed by immunoblotting. We used 10% of the lysate to detect total levels of the respective proteins; scr., scrambled. (k) Quantification of three independent experiments shows gephyrin palmitoylation levels normalized to total protein (***p<0.001 using Student's *t* test).

We next wanted to identify the primary gephyrin-palmitoylating enzyme(s). We co-expressed gephyrin-GFP together with individual HA-tagged murine DHHC enzymes in HEK293 cells and analyzed palmitoylation by ABE assays, following established protocols [16],[27]. We found that palmitoylation of gephyrin increased upon co-expression of several DHHC enzymes, with the strongest increases seen with DHHC-12, -16, -17, and -18 (Figure 5a). Accuracy of the assay was shown by the specific palmitoylation of the GABA_A_R γ2 subunit by DHHC-3 and DHHC-7 (Figure S4a) [18]. Note that steady-state expression levels of the different transfected DHHCs were uneven (Figure S4b), as was also reported by others [28],[29].

As the uneven expression pattern might misrepresent the importance of some DHHCs, and to validate the results in a more physiologically relevant environment, we additionally performed a DHHC screen in primary hippocampal neurons. As prepalmitoylated endogenous gephyrin could potentially mask the effect of transfected DHHC enzymes, we cotransfected gephyrin-GFP along with the individual DHHC enzymes. The gephyrin-GFP cluster size was quantified already 24 h posttransfection to minimize the influence of endogenous DHHC enzymes. Moreover, only neurons with adequate DHHC enzyme immunstaining were analyzed (Figure S5). We validated this approach by quantifying the size of PSD-95–GFP puncta at dendritic spines cotransfected with DHHC enzymes active (DHHC-3) or inactive (DHHC-22) towards PSD-95 [16] (Figure S6). We found that expression of DHHC-12 but none of the other DHHC family members selectively increased the cluster size of gephyrin-GFP (123.31%±8.04% of control; *n* = 2 cultures; *p* = 0.018; Figure 5b). By contrast, the density of gephyrin clusters was not affected (Figure 5d). Conversely, co-expression of gephyrin-GFP with a dominant-negative DHHS-12 construct (encoding bicistronic mCherry to visualize transfected dendrites) resulted in significantly smaller gephyrin clusters compared to controls transfected with mCherry alone (70.32%±5.69% of control; *n* = 3 cultures; *p*<0.001; Figure 5e,g). Similar to palmitoylation-deficient gephyrin, gephyrin-GFP cluster density was significantly increased in the presence of DHHS-12 (control, 5.23%±0.23%; DHHS-12, 6.08%±0.20%; *n* = 3 cultures; *p* = 0.006; Figure 5f,g). As expected, DHHC-12 overexpression did not affect the cluster size of 212,284Geph (Figure 5h). Endogenous transcripts of DHHC-12 were readily detectable in cultured neurons, consistent with relevance for palmitoylation of neuronal proteins (Figure 5i).

To confirm that endogenous DHHC-12 indeed palmitoylates gephyrin, we down-regulated the expression of DHHC-12 by means of transcript-specific cell-penetrating siRNAs in cultured neurons. As DHHC-16 was the second potent DHHC enzyme in terms of increasing gephyrin-GFP cluster size (Figure 5b), we additionally used DHHC-16–specific siRNAs to test whether this enzyme also affects palmitoylation of gephyrin. Palmitoylation of gephyrin in DHHC-12 siRNA-treated neurons was significantly decreased compared to scrambled siRNA-treated neurons (siRNA DHHC-12, 54.89%±4.11% of scrambled siRNA; *n* = 4; *p*<0.001; Figure 5j,k). DHHC-16 siRNA treatment had no effect on gephyrin palmitoylation (100.4%±7.8% of scrambled siRNA; Figure 5k), suggesting that endogenous DHHC-16 does not act directly on gephyrin in neurons. Due to the lack of appropriate antibodies, we were not able to measure endogenous DHHC protein levels after siRNA down-regulation. However, expression of HA-tagged DHHCs was significantly decreased in siRNA-treated HEK293 cells and siRNAs were delivered to virtually all neurons (Figure S7), suggesting that our approach resulted in efficient down-regulation of the respective DHHC enzyme. Importantly, palmitoylation levels of GABA_A_R γ2 and PSD-95 were not altered by knockdown of DHHC-12 and DHHC-16 (Figure 4h), which is consistent with direct effects of DHHC-12 on gephyrin clustering.

### DHHC-12–Mediated Gephyrin Palmitoylation Increases mIPSC Amplitudes

**Figure 6:**
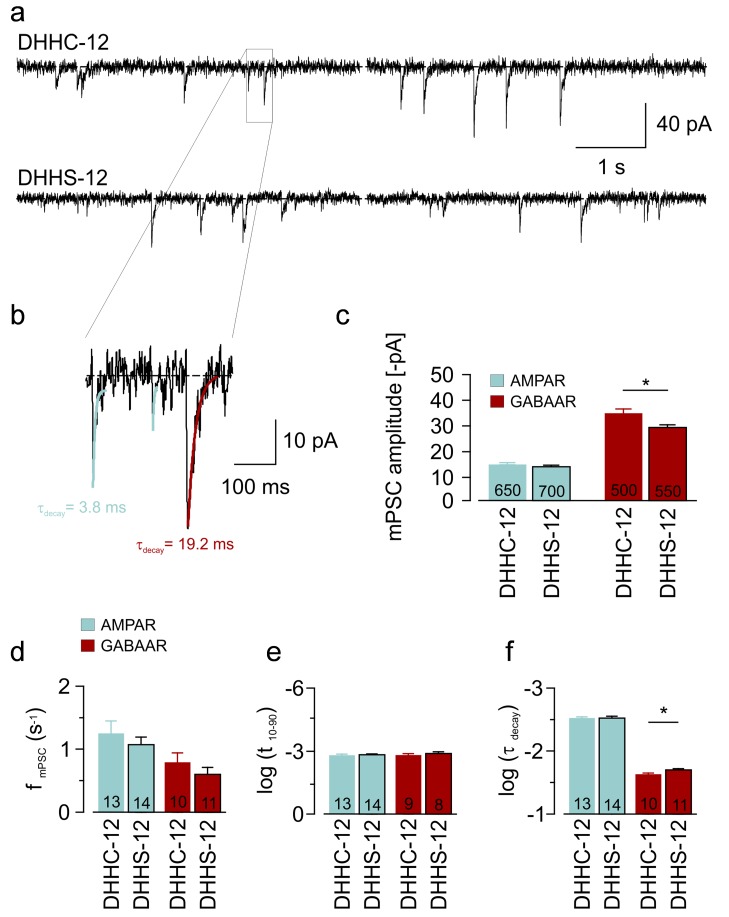
Electrophysiological analysis of GABAergic and glutamatergic mPSCs. (a) Example traces showing mPSCs recorded from DHHC-12– and DHHS-12–expressing neurons (identified using bicistronic mCherry expression). AMPAR- and GABA_A_R-mediated mPSCs were distinguished according to fast (AMPAR) and slow (GABA_A_R) decay kinetics. (b) The zoomed region of a recording from a DHHC-12–expressing neuron illustrates exponential fits of the different decay time constant (gray, AMPAR, τ = 3.8 ms; red, GABA_A_R, τ = 19.2 ms). (c–f) Quantification of amplitude, frequency, rise times (10–90), and decay time constants of AMPAR- and GABA_A_R-mediated mPSCs in DHHC-12– or DHHS-12–expressing neurons. Number of quantified events or neurons are given in parentheses.

To assess the functional impact of gephyrin palmitoylation on inhibitory synapses, we recorded miniature postsynaptic currents (mPSCs) of DHHC-12– and DHHS-12–transfected primary hippocampal neurons. AMPAR- and GABA_A_R-mediated postsynaptic currents (mEPSCs and mIPSCs) were distinguished pharmacologically as well as by their decay kinetics (see Figure 6a,b and Materials and Methods). Indeed, the amplitude of mIPSCs of DHHC-12–expressing neurons was significantly larger than in neurons that expressed the inactive DHHS-12 variant, although there were no differences in mIPSC frequency and rise times (Figure 6c–e). Thus, the data suggest that gephyrin palmitoylation increased the postsynaptic GABA_A_R pool, in agreement with the increased size of postsynaptic gephyrin clusters upon DHHC-12 expression. Furthermore, the decay time constant of mIPSCs was slightly increased in DHHC-12–expressing neurons, possibly reflecting minor changes in GABA_A_R subunit composition (Figure 6f). In contrast to mIPSCs, the properties of mEPSCs were similar with respect to all measures, suggesting that DHHC-12 affected selectively the function of proteins localized at GABAergic synapses.

### DHHC-12 Localizes to Golgi and Dendritic Shaft and Interacts with Gephyrin

**Figure 7:**
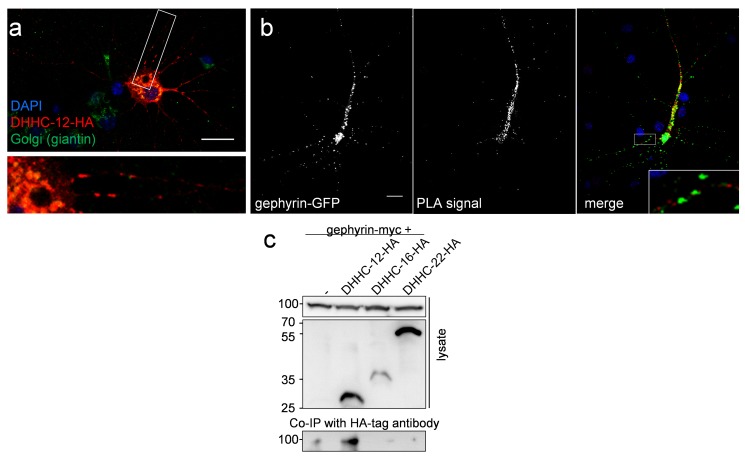
Subcellular localization of the DHHC-12/gephyrin interaction. (a) Representative image of a primary hippocampal neuron transfected with DHHC-12–HA (red) immunostained together with the Golgi-marker giantin (green). The higher magnification below shows Golgi localization of DHHC-12 in the cell body and in dendrites as vesicle-like structures. (b) PLA dots (red) demonstrate direct interaction of gephyrin-GFP and DHHC-12–HA in cultured neurons. PLA dots in the inset show gephyrin-GFP and DHHC-12–HA interaction outside gephyrin clusters. Scale bars, 20 µm. (c) Co-immunoprecipitation of gephyrin-myc from HEK293 lysates co-expressing HA-tagged DHHC enzymes as indicated with HA-tag–specific antibodies.

To assess in which neuronal cellular compartment gephyrin gets palmitoylated, we next investigated the subcellular localization of DHHC-12 and performed immunofluorescence staining of primary cultured neurons transfected with HA-tagged DHHC-12. Immunoreactivity of DHHC-12–HA showed significant overlap with staining of the Golgi-marker giantin, suggesting that the enzyme is largely localized to the Golgi (Figure 7a). In addition to the soma, DHHC-12–HA accumulated in primary dendrites with a staining pattern, suggesting its presence in vesicular Golgi outposts (Figure 7a).

We then asked whether gephyrin interacts with DHHC-12 and performed *in situ* proximity ligation assays (PLAs), which allow detection and intracellular localization of protein–protein interactions, in DHHC-12–HA and gephyrin-GFP cotransfected primary neurons (Figure 7b). Interestingly, PLA dots were found mainly in nonsynaptic regions, as indicated by close proximity but not overlapping localization of PLA dots and gephyrin clusters. Interaction of the two proteins was also verified biochemically upon heterologous co-expression in HEK293 cells. Myc-tagged gephyrin was co-immunoprecipitated by HA-tagged DHHC12, but not the controls HA–DHHC-16, HA–DHHC-22, or HA–antibody loaded beads (Figure 7c). In aggregate, we conclude that DHHC-12 acts as a principal gephyrin-palmitoylating enzyme and thereby modulates membrane association and accumulation of gephyrin at inhibitory synapses.

### Gephyrin Palmitoylation Is Regulated by GABA_A_R Activity

**Figure 8:**
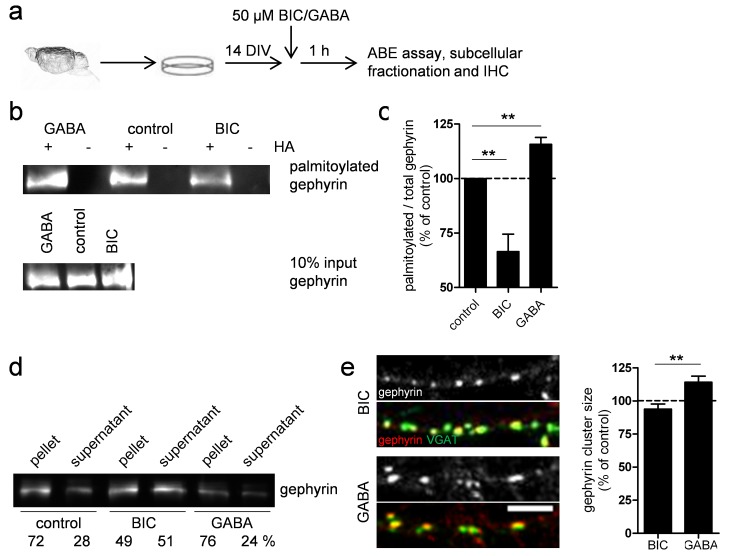
Gephyrin palmitoylation is regulated by GABA_A_R-activity. (a–e) Primary hippocampal neurons were cultured 14 DIV and exposed to 50 µM bicuculline (BIC) or GABA for 1 h. (b) Representative Western blot shows altered gephyrin palmitoylation in the presence of BIC and GABA. (c) Quantification of gephyrin palmitoylation in neurons treated as in (a). Palmitoylation was normalized to total levels of gephyrin (*n* = 3). (d) Representative immunoblot of subcellular fractionation of neurons after treatment as in (a). (e) Representative immunostaining of gephyrin and VGAT. Scale bar, 5 µm. Histogram showing quantification of gephyrin cluster size after exposure to bicuculline or GABA compared to control cultures (see a) from three independent cultures. All data are means ± SEM (**p<0.01 using Student's *t* test).

Palmitate turnover on various neuronal receptor-associated proteins has been shown to be regulated by neuronal activity [13]. Thus, we tested whether gephyrin palmitoylation is regulated by GABA_A_R activity (Figure 8a). When GABA_A_R activation was reduced by treatment with 50 µM of the GABA_A_R antagonist bicuculline, gephyrin palmitoylation was significantly decreased to 66.5%±8.0% (*p* = 0.004) of control cultures, whereas incubation with 50 µM GABA increased gephyrin palmitoylation to 115.7%±3.2% (*p* = 0.002; *n* = 3; Figure 8b,c). Consistently, association of gephyrin with membranes was decreased by bicuculline and increased by GABA treatment (Figure 8d). Moreover, the size of gephyrin clusters was significantly reduced in bicuculline-treated compared to GABA-treated cultures (93.82%±3.81% versus 114.09%±4.59% of control; *n* = 3 cultures; *p* = 0.001; Figure 8e). Collectively, these results indicate that GABA_A_R activity-dependent increases in gephyrin palmitoylation promote its postsynaptic clustering.

## Discussion

**Figure 9:**
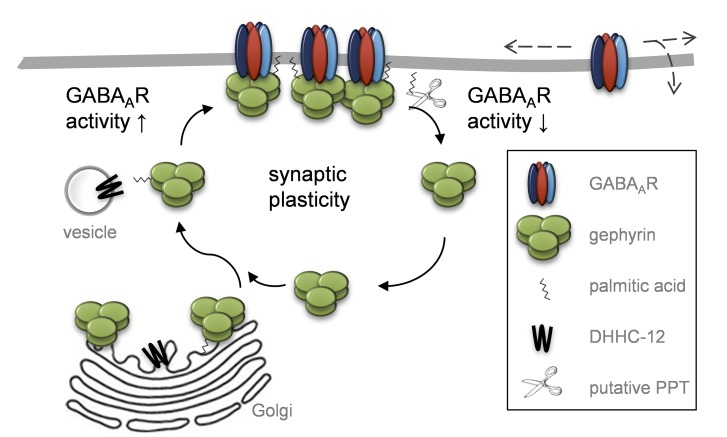
Model for palmitoylation-mediated regulation of gephyrin clustering and plasticity. Gephyrin, palmitoylated on Cys212 and 284, forms stable clusters at the postsynaptic membrane. Activity of GABAergic synapses promotes (de)palmitoylation of gephyrin by yet unknown mechanisms. Silencing of GABAergic transmission leads to gephyrin depalmitoylation and membrane release, ultimately decreasing the size of gephyrin clusters. The palmitoyl transferase DHHC-12, localized in the Golgi in neurons including presumed Golgi outposts in primary dendritic shafts, is the principle gephyrin-palmitoylating enzyme and allows dynamic (re)palmitoylation of gephyrin.

Here, we demonstrate that gephyrin is palmitoylated and describe the mechanism by which this modification modulates the function of GABAergic synapses (Figure 9). We found that postsynaptic clustering of gephyrin, an essential process for normal functioning of inhibitory synapses, is critically dependent on its palmitoylation. Lack of palmitoylation, by pharmacological inhibition or expression of palmitoylation-deficient gephyrin, led to nonsynaptic, mislocalized gephyrin clusters. Augmented palmitoylation by DHHC-12, on the other hand, increased the size of the gephyrin synaptic scaffold, thereby ultimately increasing the strength of GABAergic transmission. Together with our observation that palmitoylation is also linked to GABA_A_R activity, palmitoylation becomes a major regulatory factor in gephyrin-dependent GABAergic plasticity.

We mapped Cys212 and Cys284 within the C-domain of gephyrin as the palmitoylation sites. As a “two-lipid modification” seems to be essential for stable plasma membrane interaction [30], it is likely that within the tertiary structure of gephyrin, Cys212 and Cys284 are in close proximity. Compared to PSD-95, a smaller population of gephyrin was palmitoylated in brain. Considering a tight membrane association, the proportion of gephyrin that is palmitoylated might, however, be significantly greater at synapses. Gephyrin fulfills a second metabolic function by catalyzing the last step of the molybdenum cofactor biosynthesis, which in the brain takes place in glia cells [31]. Consequently, this fraction of metabolically active nonneural gephyrin is expected to be nonpalmitoylated and cytosolically localized. As gephyrin forms stable oligomers (hexamers and nonamers), we predict that only individual gephyrin molecules within an oligomer are palmitoylated. This idea is supported by ultrastructural data of synaptic gephyrins showing that gephyrin clusters have a nonuniform shape and are composed of certain microdomains [32]. Furthermore, immuno-EM studies with different monoclonal antibodies revealed that the gephyrin C-domain is closer to the synaptic membrane as compared to the N-terminal G-domain [33]. In contrast, each molecule of nonoligomerized proteins such as PSD-95 likely needs to be palmitoylated to physically associate with the membrane.

Gephyrin molecules exchange continuously between synaptic and nonsynaptic populations [34]. Our data suggest that palmitoylation of gephyrin is crucial for normal clustering at postsynaptic sites. We observed a significantly increased density of palmitoylation-deficient gephyrin clusters at nonsynaptic sites. Furthermore, nonpalmitoylated gephyrin was less stably associated with the membrane and more diffusively distributed in the cytoplasm. Therefore, we suggest that palmitoylation-mediated membrane association is necessary to stabilize gephyrin clusters, hence leading to dissociation into smaller clusters when gephyrin is not palmitoylated. This fits well with the significantly decreased size and total fluorescence of clusters formed by palmitoylation-deficient gephyrin mutants in neurons (Figure 3). Thus, (de)palmitoylation cycles of gephyrin might be important for merging and splitting of gephyrin clusters as part of inhibitory synapse dynamics, as suggested earlier [35].

Besides gephyrin, palmitoylation was also shown to regulate the postsynaptic accumulation of γ2-subunit–containing GABA_A_Rs [17],[18],[36]. In contrast to GABA_A_Rs, targeting of gephyrin to synapses is not necessarily palmitoylation-dependent, as evidenced by postsynaptic clustering of the 212,284Geph variant or endogenous gephyrin in 2-BP–treated cultures (Figures 2 and 3). As palmitoylation can regulate cellular localization and binding to the plasma membrane [37], rather the association and residence time of gephyrin at the postsynaptic membrane and stabilization of gephyrin clusters are palmitoylation-dependent.

Recent studies demonstrated dynamic, neuronal activity-dependent changes in gephyrin clustering *in vivo* [8],[9],[38],[39]. However, a specific molecular mechanism was not identified so far. Our study provides a novel mechanism whereby palmitoylation-dependent gephyrin clustering is linked to GABAergic transmission. Activation of GABA_A_Rs increased the palmitoylation of gephyrin and consequently its membrane affinity, whereas blockade of GABAergic transmission had the opposite effects. This GABA_A_R activity-dependent modulation of gephyrin clusters allows dynamic and structural changes of the postsynaptic architecture.

Studies on PSD-95 have identified that palmitoylation is important for glutamatergic synaptic plasticity and function [14],[40]. Mechanistically, glutamatergic transmission leads to depalmitoylation of PSD-95 and destabilization of the postsynaptic density [14]. Our study extends this view and indicates that palmitoylation of postsynaptic scaffolding proteins is a key and general factor to adjust the synaptic strength. In fact, opposing actions of GABA_A_R and glutamate receptor activation on palmitoylation of gephyrin and PSD-95, respectively, implicate that this posttranslational mechanism can serve as a common regulator of GABAergic and glutamatergic synaptic transmission.

We identified DHHC-12 as the major gephyrin-palmitoylating enzyme. The activity of DHHC-12 seems to be selective for GABAergic versus glutamatergic synapses and suggests that the enzyme is indispensable for normal inhibitory function, whereas excitatory transmission seems to be independent of DHHC-12 (Figure 5). Indeed, to our knowledge, gephyrin is the first identified substrate of DHHC-12, whereas other members of the DHHC family have been identified to palmitoylate proteins that reside at glutamatergic synapses [13]. Remarkably, neither dendritically localized palmitoyl transferases such as DHHC-2, -5, and -8 [40],[41] nor enzymes with a broad substrate specificity like DHHC-3 and -7 [18],[42],[43] were able to palmitoylate gephyrin in HEK293 cells or neurons (Figure 4). This finding suggests a very specific substrate recognition mechanism underlying gephyrin palmitoylation by DHHC-12.

In agreement with previous results from neuroblastoma cells [44], DHHC-12 was largely Golgi-localized in neurons, including presumed Golgi outposts in primary dendritic shafts. By analogy to glutamatergic neurotransmission-dependent palmitoylation of PSD-95 by dendritic DHHC-2 [15],[40], the presence of DHHC-12 in dendrites would allow dynamic gephyrin palmitoylation at synapses, which is supported by our PLA-based identification of gephyrin–DHHC-12 interactions in the vicinity of inhibitory synapses. Given that we observed residual gephyrin palmitoylation in DHHC-12 siRNA-treated cells, we cannot rule out that other DHHC enzymes can compensate for reduced DHHC-12 activity and/or use gephyrin as a substrate. However, as there are no closely related paralogues of DHHC-12 [13], substrate recognition and palmitoylation would probably follow divergent modes of action.

Interestingly, palmitoylation at Cys212 and Cys284 surrounds the previously reported gephyrin phosphorylation sites at residues Ser268 and Ser270 [10],[11]. The phospho-mimetic S268E variant shows an increased density of gephyrin clusters, whereas gephyrin phosphorylation at Ser268 was mainly identified in membrane-bound fractions. The authors concluded that the effect on gephyrin cluster density seen in Ser268 mutants is caused by the interrelation with another posttranslational modification [11]. Thus, there might be a crosstalk between gephyrin palmitoylation and phosphorylation on Ser268 or other phosphor-sites recently identified in the gephyrin C-domain [11],[22]. Additionally, some gephyrin-interacting proteins bind to gephyrin's C-domain [23]. Dynein light chain (DLC), a component of the motor protein complexes, was suggested to be involved in the subcellular localization of gephyrin [45]. The interaction with gephyrin was mapped to residues 203–214 [46]. Hence, it will be important to assess whether palmitoylation affects dynein-dependent functions on gephyrin.

Given that aberrant protein palmitoylation is implicated in a range of neurological diseases [47],[48], it will be interesting to see whether deregulated gephyrin palmitoylation contributes to pathogenesis in these disorders. In fact, the *ZDHHC12* gene is located in a region of chromosome 9 implicated in epilepsy and other neuropsychiatric disorders [49],[50]. Interestingly, gephyrin malfunction has also been linked to neuropsychiatric diseases including epilepsy [51],[52]. New probes, such as the recently introduced genetically encoded antibody that is sensitive to palmitoylation [15], could help to further shape our understanding of the molecular mechanisms of gephyrin palmitoylation in health and disease.

## Materials and Methods

### DNA Constructs

Regular enhanced green fluorescent protein (EGFP)-tagged gephyrin [53] served as the basis for all gephyrin expression constructs. Site-specific mutations were introduced by fusion PCR. DHHC expression constructs, HA-tagged PSD-95, and GST were described before [16]. For the electrophysiology experiments, DHHC12 and DHHS12 cDNAs were amplified with PCR and cloned in frame into a homemade expression vector containing a CMV promoter, the mCherry-coding DNA (derived from #631237, Clontech, Palo Alto, CA), and the sequence coding for the self-processing 2A peptide (EGRGSLLTCGDVEENPG/P) from the Thosea Asigna virus [54], which ensures faithful co-expression of bi-cistronic mRNAs and hence unambiguous identification of DHHC/S-12–expressing neurons based on mCherry fluorescence. Expression constructs for Sf9 cells have been described before [22].

### Antibodies

The following primary antibodies were used for Western blotting and diluted in TBS-Tween containing 1% dry-milk or 3% BSA: mouse anti-gephyrin (clone 3B11 cell culture supernatant, 1∶50); rabbit anti–PSD-95 (1∶500, Synaptic Systems); rabbit anti–GABA_A_-α1, guinea-pig anti–GABA_A_-α5, guinea-pig anti–GABA_A_-γ2 subunit (all 1∶500, Jean-Marc Fritschy, University of Zurich); rabbit anti–GABA_A_-γ2 (1∶500, Synaptic Systems); mouse anti–GABA_A_-β2/3 (1∶200, Millipore); rabbit anti–β-tubulin (1∶150, Santa Cruz); rabbit anti-GFP (1∶3,000, Abcam); mouse anti–HA-tag (1∶1,000, clone 12CA5, Roche); and mouse anti–myc-tag (1∶10 cell culture supernatant, clone 9E10). HRP secondary antibodies (Santa Cruz) were used in 1∶5,000 dilutions in TBS-Tween containing 1% dry-milk. Streptavidin HRP conjugates were from Cell Signaling.

The following antibodies were used for immunofluorescence and PLA assay: mouse anti-gephyrin (1∶50 cell culture supernatant, clone 3B11 [53]); rabbit anti-VGAT (1∶500, Synaptic Systems); mouse anti–PSD-95 (1∶500, Thermo Scientific); rabbit anti–GABA_A_-γ2 and α2 (1∶500, Synaptic Systems); mouse anti–HA-tag (1∶1,000, clone 12CA5, Roche); mouse anti–myc-tag (1∶10 cell culture supernatant, clone 9E10); rabbit anti-giantin (1∶1,000, Abcam); and rabbit anti-GFP (1∶1,000, Abcam). Secondary antibodies were all goat-raised Alexa Fluor 488 or 568 antibodies (Life Technologies).

### Cell Cultures and Transfections

Primary neuron cultures were cultured as described [52]. Neurons were usually transfected after 10–11 d in vitro (DIV) unless otherwise stated using Lipofectamin 2000 (Life Technologies) following the manufacturer's manual and cultured for an additional 1–3 d depending on the experiment. HEK293 cells were cultured and transfected as described previously [55].

### ABE Assay

Cultured cells or homogenized tissue was extracted in palmitoylation assay (PA) buffer (4% SDS, 50 mM Tris-HCl, pH 7.4, 5 mM EDTA) and briefly sonicated. We added 20 mM methyl methanethiosulfonate (MMTS) to the lysate and rotated it head-over-tail for 1 h at room temperature to efficiently block free thiol groups. Unreacted MMTS was removed by three sequential chloroform-methanol precipitations. The resulting protein pellet was usually resuspended in 50 µl PA buffer, mixed with 200 µl biotin buffer containing 1 M hydroxylamine, pH 7.4, 0.4 mM HPDP-biotin (Pierce), 0.2% Triton X-100, and protease inhibitor and rotated head-over-tail for 2 h at room temperate. Unreacted HPDP-biotin was removed by three chloroform-methanol precipitations. The final protein pellet was usually dissolved in 50–100 µl PA buffer containing 2% SDS and diluted 10-fold in PBS-containing protease inhibitors to result in a final SDS concentration of 0.2%. Aliquots were incubated for 30 min at room temperature to allow back-folding of the proteins and aggregates, and particles were removed by centrifugation for 1 min at 13,000 *g*; 10% volume served for the input control. The remaining supernatants were incubated with 15 µl Neutravidin beads (Pierce) for 90 min at room temperature or overnight at 4°C to precipitate biotinylated protein. Proteins were eluted from the beads with SDS loading buffer and subjected to immunoblotting.

### Click Chemistry for the Detection of Palmitoylation

Click chemistry labeling protocol was performed as previously described [56] and adopted to Sf9 cells as follows. Sf9 cells expressing 6×His-tagged gephyrin were labeled with 100 µM 17-ODYA (Cayman Chemicals) for 6 h. Cells were washed with PBS and resuspended into 400 µl of cold lysis buffer (100 mM sodium phosphate, pH 7.4, 150 mM NaCl, 1% Tritox X-100) containing protease inhibitor complete cocktail (Roche). Cells were briefly sonicated and incubated on ice for 30 min under frequent vortexing. After centrifugation of unbroken cells, the supernatant was subjected to chloroform/methanol precipitation and resuspended in 2% SDS, 50 mM Tris/Cl, 5 mM EDTA, pH 7.4. Click chemistry reaction began with 23 µl of cell lysates containing ∼2 mg/ml protein, 0.5 µl 5 mM biotin-PEG-N3 prepared in DMSO, 0.5 µl of 50 mM Tris(2-carboxyethyl)phosphine hydrochloride prepared freshly, and 0.5 µl 10 mM Tris[(1-benzyl-1*H*-1,2,3-triazol-4y1)methyl-1]amine prepared in DMSO/t-butanol 1∶4 v/v; was vortexed for 5 s, followed by adding 0.5 µl 50 mM CuSO_4_/5H_2_0 (freshly prepared); was vortexed again; and was incubated for 1 h at room temperature in the dark. All chemicals were from Sigma. Affinity purification of biotinoylated proteins with Neutravidin beads was performed as described for the ABE assay.

### Immunostaining and Image Analysis

Neurons 11–14 DIV were fixed with 4% paraformaldehyde in PBS for 15 min and washed in 50 mM NH_4_Cl_2_ in PBS. Unspecific binding sites were blocked with blocking solution (2% BSA, 10% goat serum, 0.2% Triton) for 1 h, and primary antibodies were applied for 1 h in blocking solution. After three additional washing steps in PBS, neurons were incubated with secondary antibodies in 2% BSA/0.2% Triton for 1 h, washed extensively with PBS, and slides were mounted on coverslips with Fluoro gel II containing DAPI (Science Service). Images were taken with a Nikon AZ-C2^+^ confocal laser scanning microscope as a z-stack of three optical sections with 0.5 µm step size. Maximum intensity projections were created and analyzed using the Nikon NIS Elements 3.2 software. Usually two 20×5 µm region of interests (ROIs) per neuron were placed on dendrites and clusters were counted using the analyze particles option in NIS Elements. Selected limitations included a cluster size that needed to be between 0.09 µm^2^ and 2 µm^2^ in size.

To determine co-localization, the size of all clusters was increased by one pixel, and co-localization was analyzed using the corresponding program routine in NIS Elements. An overlapping signal from the increased gephyrin and VGAT clusters was considered as a gephyrin cluster opposed to presynaptic terminals.

For statistical analysis, values of individual neurons were averaged and mean values were compared for significance using Student's *t* test. Pseudocolor images were created using the appropriate option in ImageJ.

### Electrophysiological Recordings

Primary hippocampal neurons were transfected on DIV6 using Effectene transfection reagent (Qiagen). Either a plasmid containing mCherry-2A–DHHC-12 or mCherry-2A–DHHS-12 was expressed for 3–4 d. An EPC-7 amplifier and Patchmaster software (HEKA) were used for patch clamp recordings. Patch pipettes, made from borosilicate glass (Science Products), had resistances of 2–6 MΩ when filled with the intracellular solution containing (in mM): KCl (130), NaCl (5), CaCl_2_ (0.5), MgCl_2_ (1), EGTA (5), and HEPES (30). The standard extracellular solution (pH 7.4) contained (in mM): NaCl (140), KCl (5), MgCl_2_ (1), CaCl_2_ (2), HEPES-NaOH (10), and glucose (10). Cells were clamped at a potential of −50 mV. Series resistances (R_s_), monitored by −5 mV voltage pulses (50 ms) applied every 5 s, were between 5 MΩ and 30 MΩ. Experiments with a more than 25% change in R_s_ throughout the recording were discarded. Data were acquired with a sampling rate of 10 kHz after filtering at 2.8 kHz. Transfected cells were identified using mCherry fluorescence. mPSCs were isolated pharmacologically by blocking NMDA receptors with DL-aminophosphonovaleric acid (APV, 100 µm; Sigma), glycine receptors with strychnine (1 µM; Sigma), and action potentials with tetrodotoxin (TTX, 1 µM; Sigma). Analyses of the mPSCs were performed with an in-house software written in IGOR 6.32A (WaveMetrics) by M. Semtner. Extracted parameters for each PSC were time of occurrence, maximal peak amplitude, 10%–90% rise time, and tau value of the single exponential fit of the decay (τdecay). As described in [57], mPSCs were divided into mEPSCs and mIPSCs according to their τdecay values—that is, mPSCs with τdecay ≤8 ms were classified as mEPSCs and those with τdecay >8 ms as mIPSCs. Current traces (1 to 5 min long) from 16 different recorded neurons were analyzed for each condition (DHHC-12 or DHHS-12). We quantified 50 mEPSCs and 50 mIPSCs per recorded neuron. The frequency of occurrence and the logarithmical values of the rise time and τdecay were normally distributed. Thus, the bar graphs show the mean ± SEM of the averaged values from the cells, and differences were tested with the Student's *t* test. However, since the amplitude values of the mEPSCs and mIPSCs were not normally distributed, mean ± SEM of the median values extracted from each cell is shown, and the difference between the two groups was tested by performing the Kruskal–Wallis test.

### Pharmacological Treatment of Hippocampal Neurons

The following substances were used for pharmacological manipulation: 50 µM bicuculline (Calbiochem) from a 1,000× stock prepared in ddH_2_0. For GABA_A_R activation, neurons were incubated in medium containing freshly prepared 50 µM GABA (Invitrogen) from a 100× stock in H_2_0. For protease inhibition, leupeptin and pepstatin (both 10 mg/l, Sigma) were added to the neurons. 2-Bromopalmitic acid (Sigma) was diluted 1∶1,000 from the stock solution (50 mM in ethanol or DMSO). Ethanol and DMSO were added to the control neurons. For downstream Western blotting, neurons were dissolved directly in 2× SDS loading buffer.

### Mass Spectrometry

#### Tryptic in-solution digestion

Proteins were subjected to tryptic in-solution digestion within a filtration device according to the Filter Aided Sample Preparation (FASP) procedure [58] but without reduction and carbamidomethylation of cysteine residues to retain modification with Biotin-HPDP and MMTS. Prior to nano-LC-MS/MS analysis, the peptides were desalted using STAGE Tip C18 spin columns (Proxeon/Thermo Scientific) as described elsewhere [59]. Eluted peptides were concentrated in vacuo and then resuspended in 0.5% acetic acid in water.

#### Nano-LC ESI-MS/MS

Analyses using reversed phase liquid chromatography coupled to nano-flow electrospray tandem mass spectrometry were carried out using an EASY nLC II nano-LC system (Proxeon/Thermo Scientific) with a 150 mm C18 column (internal diameter, 75 µm; Dr. Maisch GmbH) coupled to a LTQ/Orbitrap mass spectrometer (LTQ Orbitrap Discovery, Thermo Scientific). Peptide separation was performed at a flow rate of 250 nl/min over 79 min (10% to 40% in 60 min): buffer A, 0.1% formic acid in H_2_O; buffer B, 0.1% formic acid in acetonitrile). Survey full scan MS spectra (m/z 350 to 2000) of intact peptides were acquired in the Orbitrap at a resolution of 30,000 using m/z 445.12003 as a lock mass. The mass spectrometer acquired spectra in “data dependent mode” and automatically switched between MS and MS/MS acquisition. Signals with an unknown charge state and +1 were excluded from fragmentation. The 10 most intense peaks were isolated and automatically fragmented in the linear ion trap using collision-induced dissociation (CID). To select specific peptides of interest for fragmentation, the samples were measured again using a list of parent masses.

Sequest as implemented in the Proteome Discoverer 1.3 software (Thermo Scientific) was used for protein identification by searching the database of *Spodoptera frugiperda* using oxidation at methionine residues, Biotin-HPDP (+428.192 Da) and MMTS (+45.988 Da) at cysteine residues, and phosphorylation at serine, threonine, and tyrosine residues as variable modifications. Mass tolerance for intact peptide masses was 10 ppm for Orbitrap data and 0.8 Da for fragment ions detected in the linear trap. Search results were filtered to contain only high confident rank 1 peptides (false discovery rate ≤1%) with a mass accuracy of ≤5 ppm and a peptide length of ≥6 amino acid residues. Peptides had to match score versus charge state criteria (2.0 for charge state 2, 2.25 for charge state 3, and 2.5 for charge state 4).

### Expression and Purification of Gephyrin from Sf9 Cells

Recombinant 6×His-tagged gephyrin was expressed in Sf9 cells and affinity-purified using a nickel-nitrilotriacetic acid resin (Ni-NTA) as described previously [22] with minor modifications. The lysis buffer contained phosphate-buffered saline buffer (PBS), 10 mM imidazole, and 1% Triton X-100, and lysed cells were incubated for 30 min on ice before pelleting cell debris.

### Western Blotting and Statistical Analysis

Proteins were separated by SDS-PAGE (6% to 12% acrylamide) and immunoblotted using standard protocols. Membranes were probed with horseradish peroxidase or alkaline phosphatase-conjugated secondary antibodies. Detection was carried out either using AP-reaction or using chemiluminescence and the ECL system with a cooled CCD camera (Decon). Band intensities were quantified using ImageJ (http://rsb.info.nih.gov/ij/). Images were converted to 8-bit, and background was reduced using the “subtract background” option. Desired bands were selected manually with the freehand function, and the enclosed areas were automatically analyzed by the software.

### PLA

At 24 h after transfection of hippocampal neurons, PLA was carried out using Duolink II detection kit (Olink Bioscience). Blocking and incubation of primary antibodies and mouse and rabbit secondary probes were performed as described in the immunostaining section. PLA ligation and amplification of PLA dots was performed following the manufacturer's instructions. The following antibodies have been used: mouse anti-gephyrin (clone 3B11) and rabbit anti-GFP (Santa Cruz). Coverslips were mounted on glass slides using Fluoro gel II mounting medium and visualized by fluorescence microscopy.

### Co-Immunoprecipitation

Protein extracts were prepared in IP buffer (50 mM Tris, pH 7.4, 25 mM NaCl, 1% Triton X-100, protease and phosphatase inhibitor cocktail (Roche)). The postnuclear fraction usually containing 100 µg to 1 mg of protein was incubated with protein-A/G Sepharose beads (Santa Cruz) preloaded with HA-tag–specific antibodies and incubated with the extracts for 2 h at room temperature. After a brief centrifugation (500× *g*, 2 min), the immune beads were washed two times with IP buffer. The adsorbed proteins were eluted from the immune beads by boiling in 50 µl of SDS loading buffer. Immunoprecipitated samples were subjected to SDS-PAGE followed by immunoblotting.

### siRNA-Mediated Knockdown of DHHCs

Accell siRNA (Thermo Scientific) was used to knock down DHHC enzymes in primary hippocampal neurons. The siRNA is modified for use without a transfection reagent and passively penetrates the cells. SMARTpool siRNAs containing four specific siRNAs were used to knock down DHHC-12 and DHHC-16 and siRNA Control Kit–Green to proof uptake efficiency by fluorescence microscopy and toxicity to the cells. Efficient knockdown of DHHC enzymes was tested in HEK293 cells by transfection with DHHC-12 or DHHC-16 vectors and incubation of the cells in the presence of 0.5 µM siRNAs for 24 h. Steady-state levels of the DHHC enzymes were subsequently analyzed by Western blotting. Primary hippocampal neurons at DIV 6 were supplemented with Accell siRNA at a final concentration of 0.5 µM and grown for 3 d. Culture medium was exchanged to normal growth medium without siRNAs, and neurons were grown for another 48 h. By this we ensured a high uptake efficiency (>90% of control neurons showed green fluorescence) and viability of neurons.

### Detergent Solubilization and Sucrose Gradient

A brain of an adult mouse was cut into small cubes and resuspended in cold HBS buffer (10 mM Tris, pH 7.4, 5 mM EDTA, 320 mM sucrose, protease inhibitor, 1 mM sodium orthovanadate, 1 mM sodium fluoride) and homogenized. Postnuclear fraction was centrifuged at 16.000× *g* for 15 min (4°C), and the pellet was resuspend in 700 µl TNE buffer (50 mM Tris pH 7.4, 150 mM NaCl, 5 mM EDTA). The lysate was briefly sonicated, split into two equal halves, and proteins were extracted using either TNE buffer containing 0.5% Triton X-100 or Triton X-100 supplemented 0.5% saponin in a final volume of 1.5 ml. The solubilized brain lysates were mixed with 80% sucrose to result in a 40% sucrose solution and pipetted in a 10 ml ultracentrifugation tube. Brain lysates were overlaid stepwise with a 5 ml 25% sucrose and 2 ml 5% sucrose solution containing 0.05% Triton X-100 or additionally 0.05% saponin. Samples were ultracentrifuged in a swing-out rotor with 150.000× *g_av_* for 14 h. After centrifugation, the sucrose gradient was fractionated in 1 ml steps from top to bottom and the same volumes of protein were resolved in an SDS-PAGE.

### Enrichment of Synaptosomes

One brain from an adult mouse was washed in ice-cold homogenization buffer (HB; 320 mM sucrose, 4 mM HEPES, pH 7.4, protease inhibitor cocktail (Roche)). The washed brain was cut into small pieces with a scalpel, and tissue was further disrupted using a motor-driven homogenizer at 900 rpm/10 strokes (PotterS from Sartorius) in 12 ml HB. Cell debris and intact cells were removed by centrifugation at 800× *g* for 5 min. The supernatant (S1 fraction) was further centrifuged at 12,000× *g* for 15 min and the resulting pellet (P1) homogenized in 12 ml HB. The resuspended pellet was centrifuged for 15 min at 14,500× *g*. The pellet (P2) contains enriched synaptosomes and was homogenized in buffers depending on the downstream applications.

The numerical data used in all figures are included in Data S1.

## Supporting Information

Data S1 Excel spreadsheet containing, in separate sheets, the underlying numerical data and statistical analysis for Figure panels 2b, 2c, 2e, 3d, 3e, 4b, 4c, 4e, 4h, 4h′, 5b, 5d, 5e, 5f, 5h, 5k, 6c, 6d, 6e, 6f, 8c, and 8f.
(XLSX)

Figure S1 Inhibition of palmitoylation leads to decreased surface-expressed levels of synaptic GABA_A_R subunits. (a) Primary hippocampal neurons from control and 2-BP–treated cultures were surface labeled with a primary amine reactive biotin reagent. (b) Upon Neutravidin beads purification, indicated GABA_A_R receptor subunits were visualized and quantified on a Western blot level. The quantification reveals a significant surface reduction of all synaptic subunits, whereas the extrasynaptic α5 subunit is not affected. Notably, reduction of GABA_A_R α1 and β2/3 subunit surface expression mirrored the reduction of gephyrin cluster size (see Figure 2B). Loading controls show the overall steady-state levels of the proteins, which were not changed for GABA_A_R subunits. Gephyrin steady-state levels were substantially reduced after 2-BP treatment. All data are means ± SEM (GABA_A_R subunits, β2/3, 72.29%±3.73%; α1, 72.4%±0.79%; γ2, 36.92%±4.45%; α5, 104.0%±4.0% of control; **p<0.01, ***p<0.001 using Student's *t* test; *n* = 3).
(TIF)

Figure S2 Oligomerization of gephyrin is not palmitoylation-dependent. Size exclusion chromatography was performed with control and 2-BP–treated lysates of primary hippocampal neurons. Fifteen fractions were collected and analyzed by immunoblot with gephyrin and PSD-95 antibodies. Corresponding elution fractions of reference proteins are shown above the immunoblots. Gephyrin (83 kDa) was eluted at a size of native hexamers and nonamers, whereas PSD-95 eluted at its monomeric size. Inhibition of palmitoylation did not change oligomerization of gephyrin or PSD-95.
(TIF)

Figure S3 Gephyrin-GC is palmitoylated on Cys212 and Cys284. Immunoblots show GFP-tagged gephyrin-GC domains and cysteine-to-serine variants that have been expressed in HEK293 cells and analyzed by ABE. Input served as loading controls of the individual mutants. Gephyrin is palmitoylated on Cys212 and Cys284, as shown by the 212,284GephGC band intensity that does not exceed the –HA internal control.
(TIF)

Figure S4 Co-expression of individual 23 DHHC enzymes with GABA_A_R γ2 in HEK-293 cells and steady-state levels of individual DHHC enzymes upon overexpression. (a) Individual HA-tagged DHHC constructs or GST-HA as control (–) were co-transfected with myc-tagged GABA_A_R γ2 for 24 h in HEK293 cells and analyzed with the ABE assay. Omitting HA demonstrated specificity of the assay. Palmitoylation of the γ2 subunit is specifically increased by DHHC-3 and DHHC-7 [1] and confirms the specificity and accuracy of our experimental setup. (b) Western blot of the lysates revealed expression of all DHHCs, although at variable levels. Asterisks in (B) show nonspecific cross-reacting protein bands.
(TIF)

Figure S5 Representative images of primary hippocampal neurons co-transfected with gephyrin-GFP and individual HA-tagged DHHC enzymes. Only images of neurons with adequate immunostaining of the individual DHHC enzymes were taken and further analyzed. Scale bar, 20 µm.
(TIF)

Figure S6 PSD-95–GFP puncta size and intensity increase in the presence of expressed DHHC-3. (a) Representative images of PSD-95–GFP fluorescence in control or DHHC-3–HA co-expressing primary hippocampal neurons. Scale bar, 10 µm. (b) Laser-scanning microscopy was used to acquire neuronal images, and dendritic PSD-95 clusters were quantified. Data are means ± SEM (PSD-95 puncta size, control, 0.28±0.02 µm^2^; DHHC-3, 0.35±0.02 µm^2^; DHHC-22, 0.27±0.03 µm^2^; **p<0.01 using Student's *t* test). Two independent cultures were used for the quantification.
(TIF)

Figure S7 Specific siRNAs efficiently down-regulate the expression of DHHC-12 and DHHC-16. (a) Immunoblots of DHHC-12–HA or DHHC-16–HA expressed in HEK293 cells in the presence of scrambled or DHHC-specific siRNAs; β-tubulin served as loading control. The palmitoyl transferases are efficiently knocked down by the specific siRNAs. (b) Representative images show efficient penetration of primary hippocampal neurons by the fluorescent scrambled siRNAs after 7+3 DIV. DAPI staining indicated that siRNA penetration is not toxic to the neurons.
(TIF)

Text S1 Supporting methods.
(PDF)

## Abbreviations

17-ODYA: 17-octadecynoic acid
2-BP: 2-bromopalmitate
ABE: Acyl-biotin exchange
DRM: detergent-resistant membrane
GABA_A_Rs: γ-aminobutyric acid type A receptors
mI/EPSC: miniature inhibitory/excitatory postsynaptic current
PLA: proximity ligation assay
VGAT: vesicular GABA transporter
