# Supplementary figures and images for: Palmitoylation of Gephyrin Controls Receptor Clustering and Plasticity of GABAergic Synapses
Source: PLoS Biol. 2014 Jul 15;12(7):e1001908. doi: 10.1371/journal.pbio.1001908 (PMC4099074; doi:10.1371/journal.pbio.1001908)

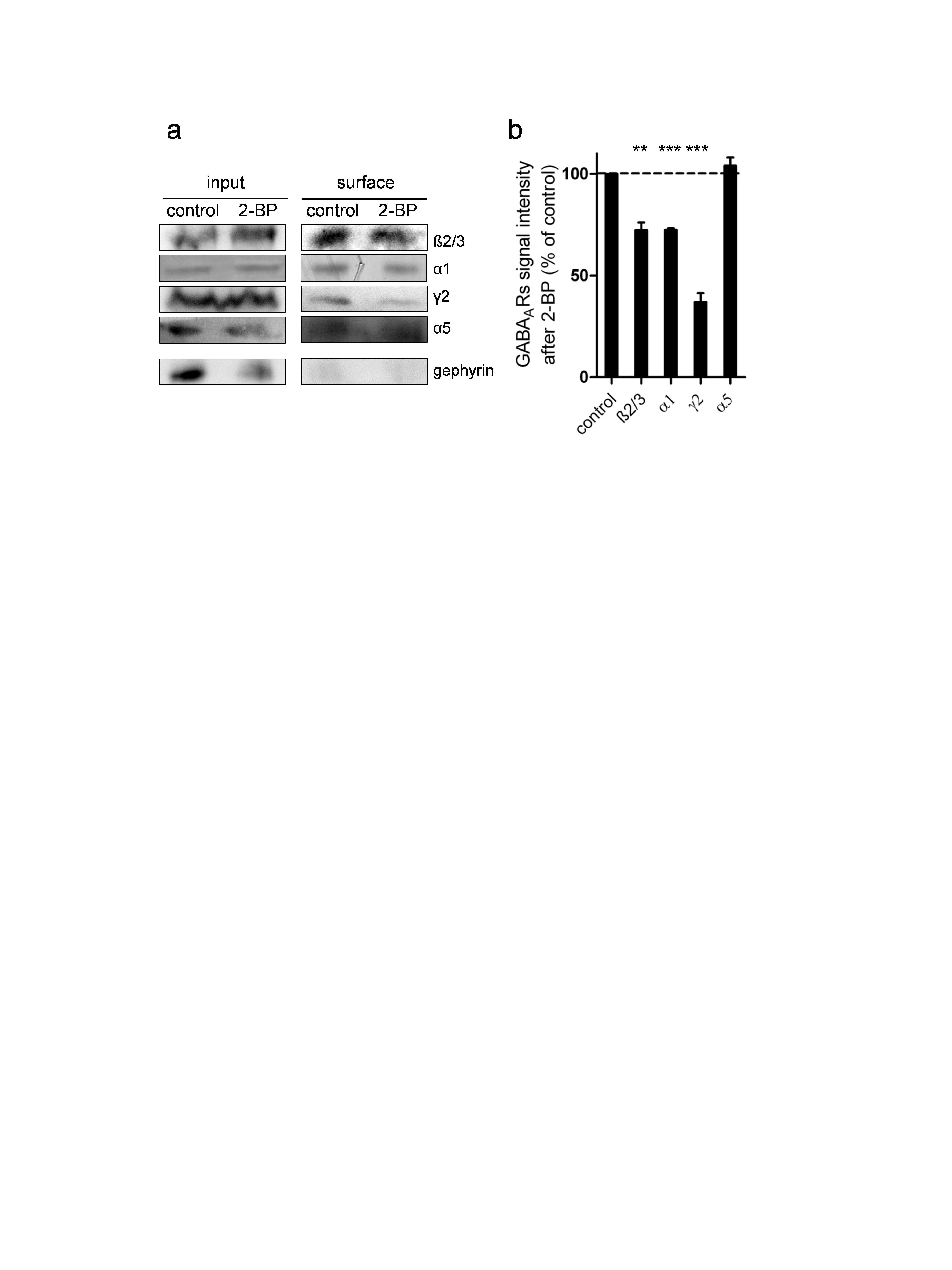

Supplement: Figure S1 — Inhibition of palmitoylation leads to decreased surface-expressed levels of synaptic GABAAR subunits. (a) Primary hippocampal neurons from control and 2-BP–treated cultures were surface labeled with a primary amine reactive biotin reagent. (b) Upon Neutravidin beads purification, indicated GABAAR receptor subunits were visualized and quantified on a Western blot level. The quantification reveals a significant surface reduction of all synaptic subunits, whereas the extrasynaptic α5 subunit is not affected. Notably, reduction of GABAAR α1 and β2/3 subunit surface expression mirrored the reduction of gephyrin cluster size (see Figure 2B). Loading controls show the overall steady-state levels of the proteins, which were not changed for GABAAR subunits. Gephyrin steady-state levels were substantially reduced after 2-BP treatment. All data are means ± SEM (GABAAR subunits, β2/3, 72.29%±3.73%; α1, 72.4%±0.79%; γ2, 36.92%±4.45%; α5, 104.0%±4.0% of control; **p<0.01, ***p<0.001 using Student's t test; n = 3). (TIF) [file pbio.1001908.s002.tif]

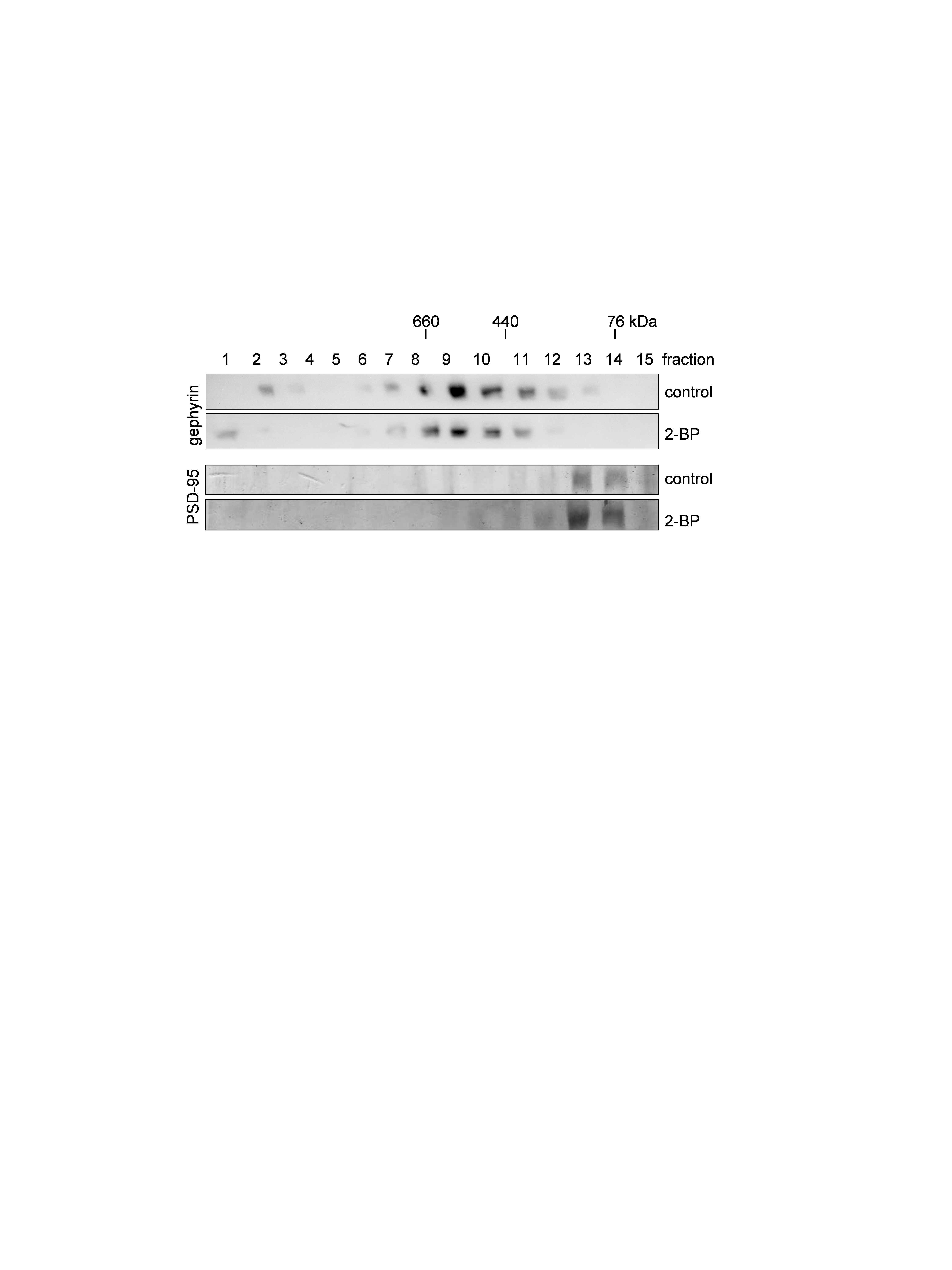

Supplement: Figure S2 — Oligomerization of gephyrin is not palmitoylation-dependent. Size exclusion chromatography was performed with control and 2-BP–treated lysates of primary hippocampal neurons. Fifteen fractions were collected and analyzed by immunoblot with gephyrin and PSD-95 antibodies. Corresponding elution fractions of reference proteins are shown above the immunoblots. Gephyrin (83 kDa) was eluted at a size of native hexamers and nonamers, whereas PSD-95 eluted at its monomeric size. Inhibition of palmitoylation did not change oligomerization of gephyrin or PSD-95. (TIF) [file pbio.1001908.s003.tif]

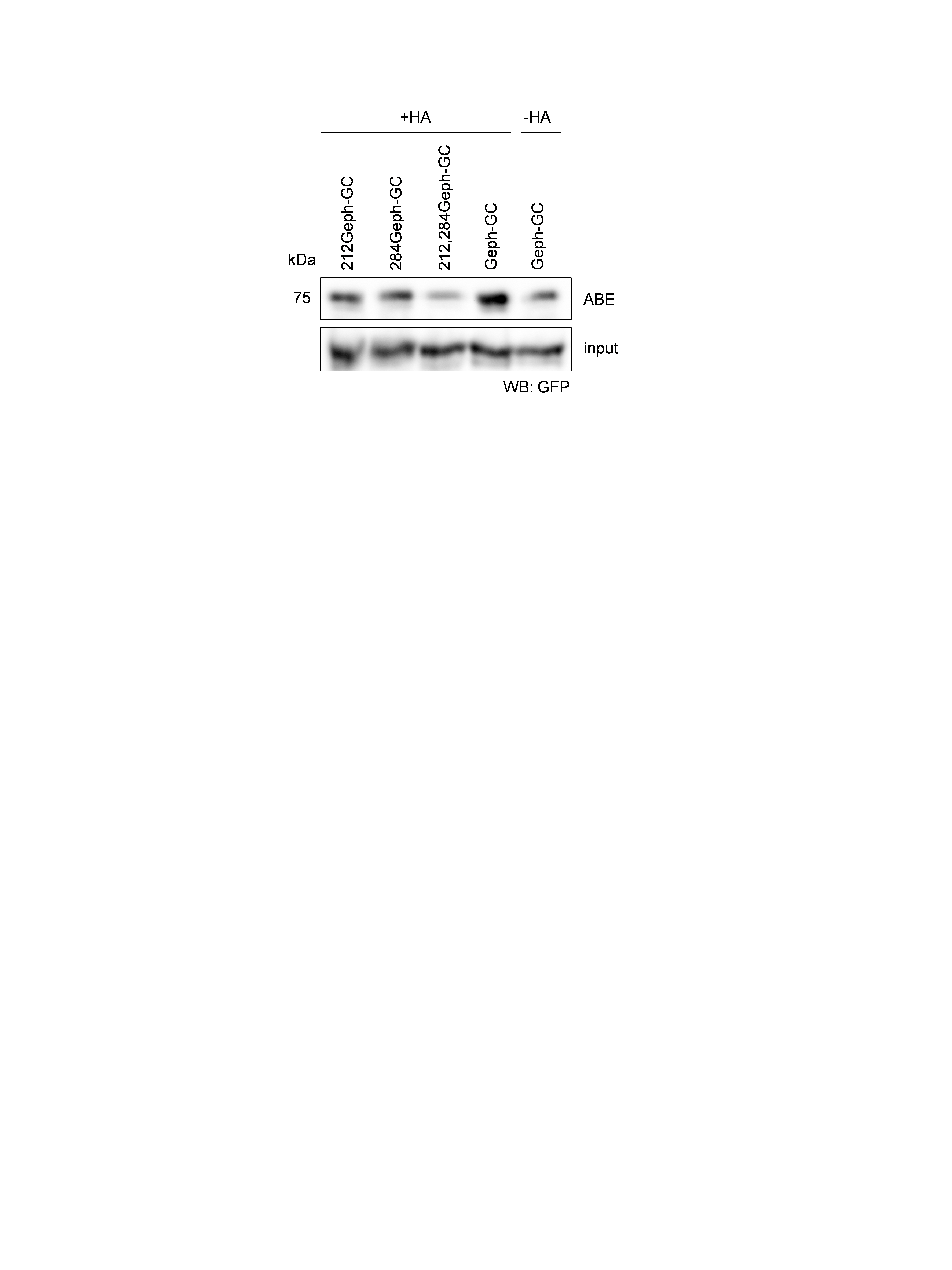

Supplement: Figure S3 — Gephyrin-GC is palmitoylated on Cys212 and Cys284. Immunoblots show GFP-tagged gephyrin-GC domains and cysteine-to-serine variants that have been expressed in HEK293 cells and analyzed by ABE. Input served as loading controls of the individual mutants. Gephyrin is palmitoylated on Cys212 and Cys284, as shown by the 212,284GephGC band intensity that does not exceed the –HA internal control. (TIF) [file pbio.1001908.s004.tif]

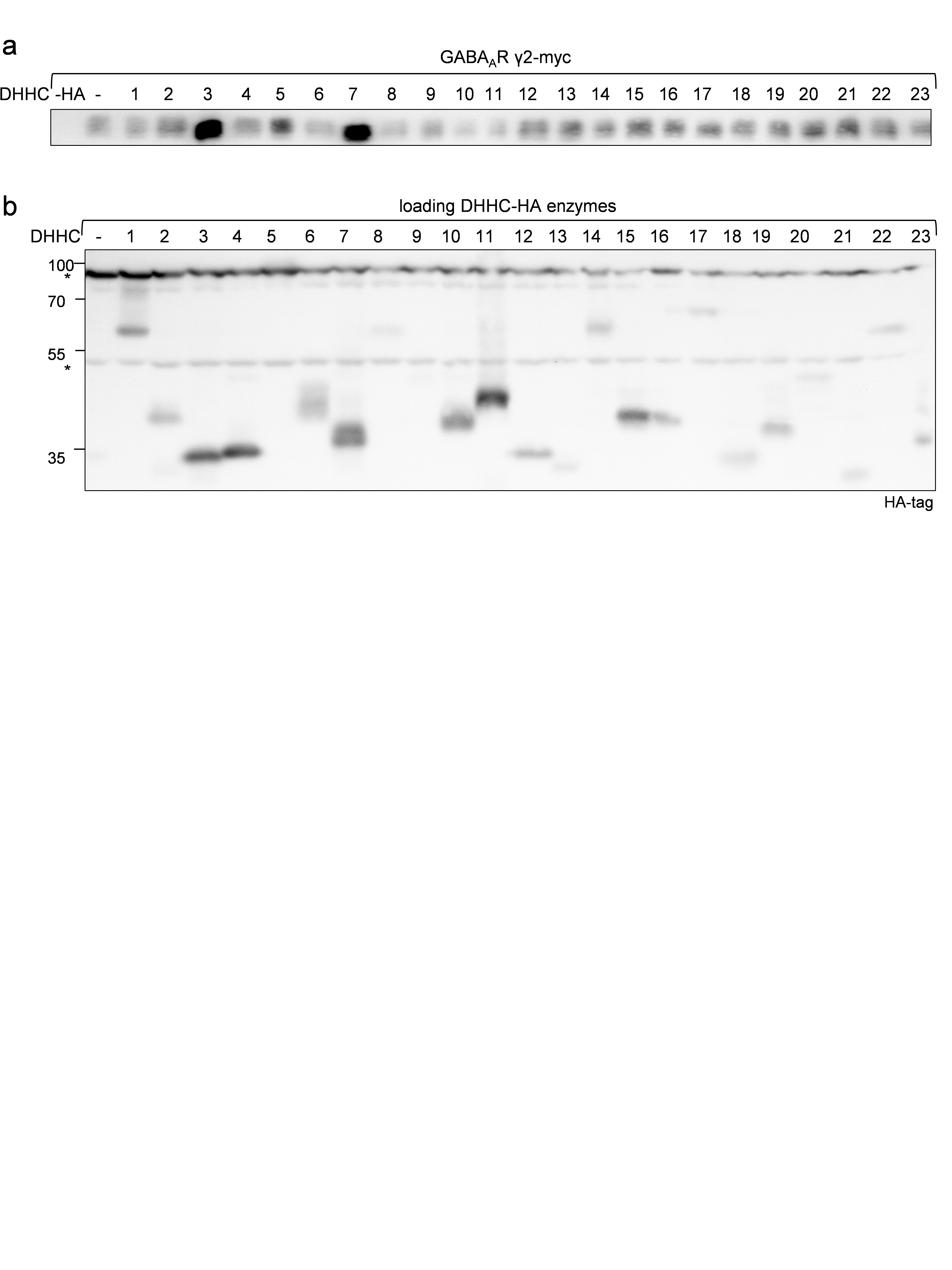

Supplement: Figure S4 — Co-expression of individual 23 DHHC enzymes with GABAAR γ2 in HEK-293 cells and steady-state levels of individual DHHC enzymes upon overexpression. (a) Individual HA-tagged DHHC constructs or GST-HA as control (–) were co-transfected with myc-tagged GABAAR γ2 for 24 h in HEK293 cells and analyzed with the ABE assay. Omitting HA demonstrated specificity of the assay. Palmitoylation of the γ2 subunit is specifically increased by DHHC-3 and DHHC-7 [1] and confirms the specificity and accuracy of our experimental setup. (b) Western blot of the lysates revealed expression of all DHHCs, although at variable levels. Asterisks in (B) show nonspecific cross-reacting protein bands. (TIF) [file pbio.1001908.s005.tif]

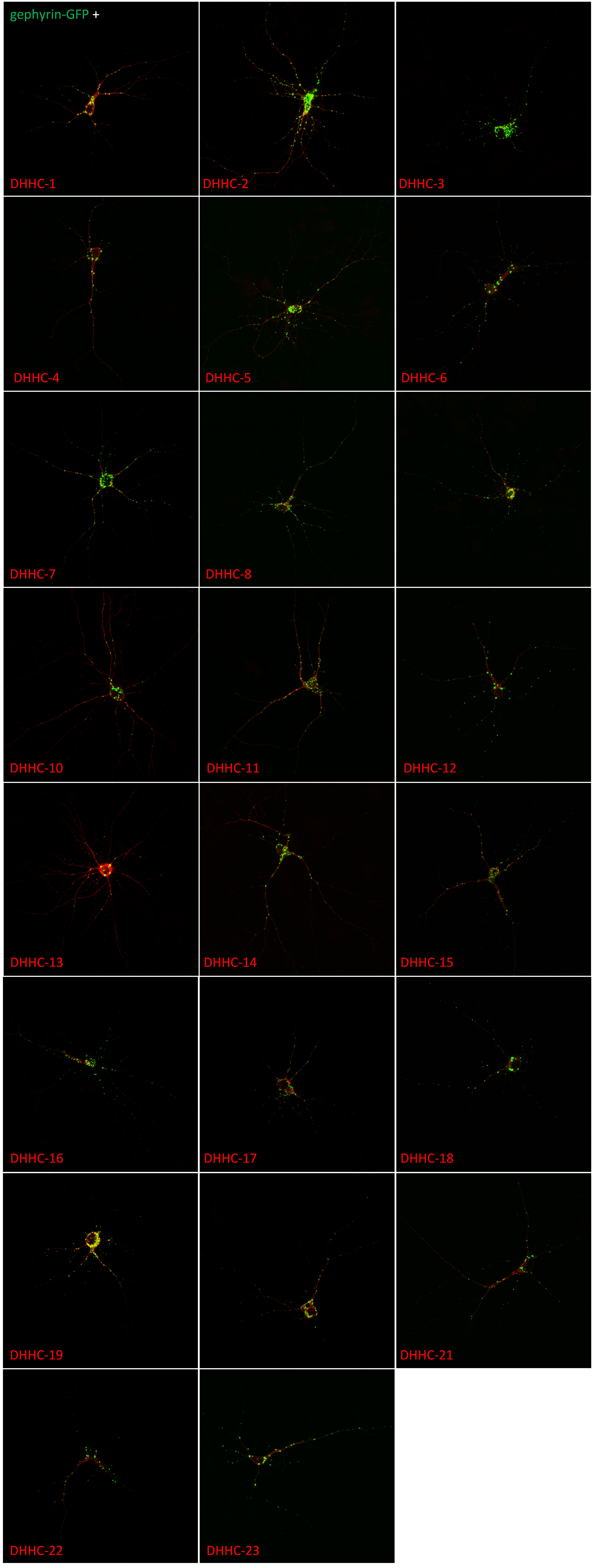

Supplement: Figure S5 — Representative images of primary hippocampal neurons co-transfected with gephyrin-GFP and individual HA-tagged DHHC enzymes. Only images of neurons with adequate immunostaining of the individual DHHC enzymes were taken and further analyzed. Scale bar, 20 µm. (TIF) [file pbio.1001908.s006.tif]

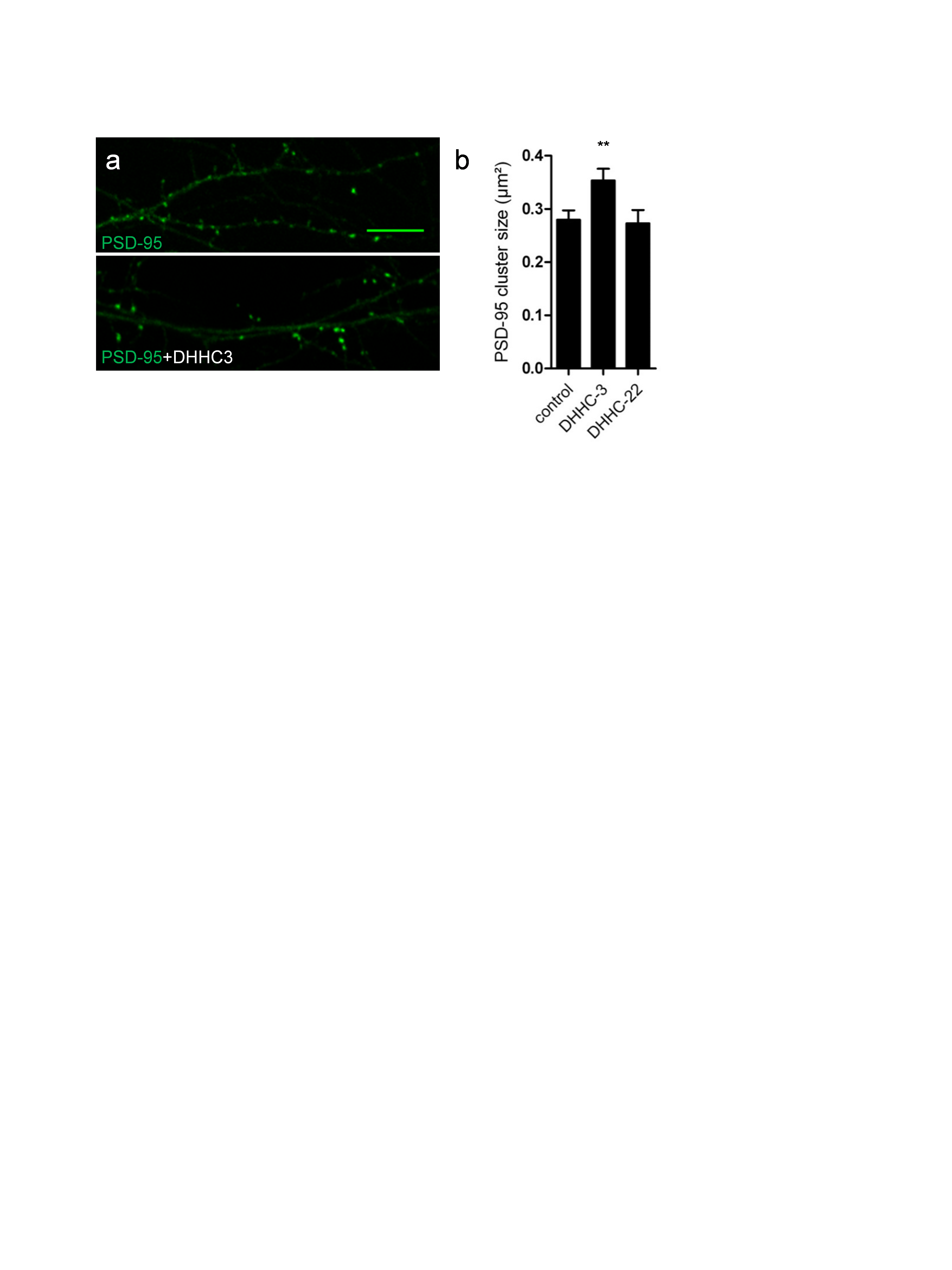

Supplement: Figure S6 — PSD-95–GFP puncta size and intensity increase in the presence of expressed DHHC-3. (a) Representative images of PSD-95–GFP fluorescence in control or DHHC-3–HA co-expressing primary hippocampal neurons. Scale bar, 10 µm. (b) Laser-scanning microscopy was used to acquire neuronal images, and dendritic PSD-95 clusters were quantified. Data are means ± SEM (PSD-95 puncta size, control, 0.28±0.02 µm2; DHHC-3, 0.35±0.02 µm2; DHHC-22, 0.27±0.03 µm2; **p<0.01 using Student's t test). Two independent cultures were used for the quantification. (TIF) [file pbio.1001908.s007.tif]

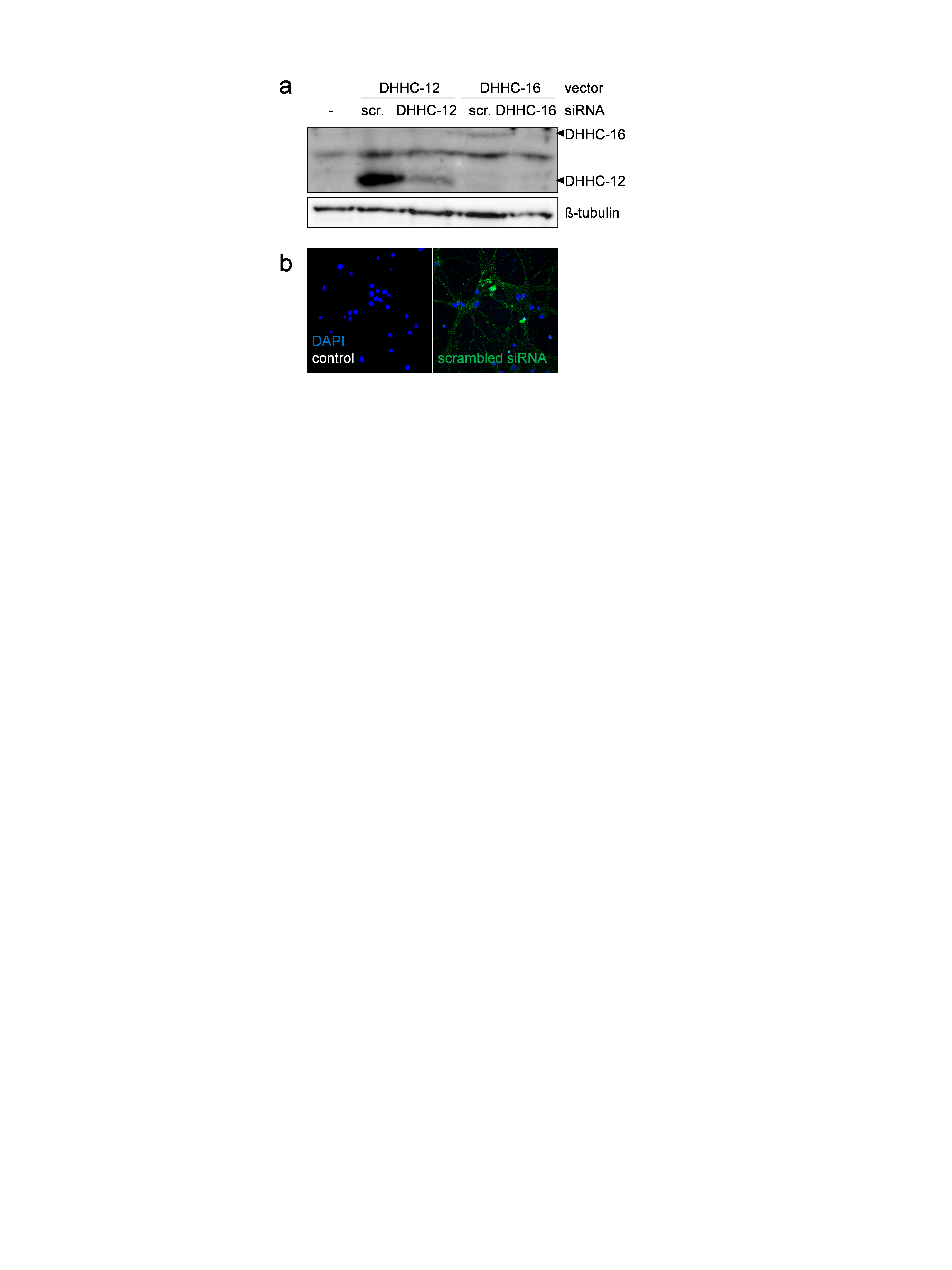

Supplement: Figure S7 — Specific siRNAs efficiently down-regulate the expression of DHHC-12 and DHHC-16. (a) Immunoblots of DHHC-12–HA or DHHC-16–HA expressed in HEK293 cells in the presence of scrambled or DHHC-specific siRNAs; β-tubulin served as loading control. The palmitoyl transferases are efficiently knocked down by the specific siRNAs. (b) Representative images show efficient penetration of primary hippocampal neurons by the fluorescent scrambled siRNAs after 7+3 DIV. DAPI staining indicated that siRNA penetration is not toxic to the neurons. (TIF) [file pbio.1001908.s008.tif]
