# Supplementary material for: Palmitoylation of Gephyrin Controls Receptor Clustering and Plasticity of GABAergic Synapses
Source: PLoS Biol. 2014 Jul 15;12(7):e1001908. doi: 10.1371/journal.pbio.1001908 (PMC4099074; doi:10.1371/journal.pbio.1001908)
Supplement: Text S1 — Supporting methods. (PDF) [file pbio.1001908.s009.pdf]

Supporting Methods

RNA extraction and semi-quantitative PCR

RNA was extracted using TRI Reagent (Sigma) following the manufacture's protocol. cDNA was synthesized using Superscript III (Invitrogen) that contained all solutions mentioned in the following: RNA (2 µg) was mixed with 1 µl primer Qt and 1 µl dNTPs and water in a final volume of 13.5 µl. After heating at 65 °C for 5 min and cooling on ice, the mixture was added to a PRC tube containing 4 ml Superscript III buffer, 1 µl DTT and 0.5 µl Superscript III enzyme. cDNA was amplified by PCR using the following temperature cycle: 25 °C, 5 min; 55 °C, 60 min; 70 °C 15 min.1 µl of cDNA products were amplified with 1x redTaq-mix (Invitrogen) in the presence of specific primers for Gephyrin, together with GAPDH primers used as an internal control. The conditions were chosen so that none of the RNAs analyzed reached a plateau at the end of the amplification protocol, i.e. they were in the exponential phase of amplification, and that the two sets of primers used in each reaction did not compete with each other. Primers used: Gapdh for (306-325) 5'-aggccggtgctgagtatgtc-3'; Gapdh rev (835-816) tgcctgcttcaccaccttct; Gphn for (1921-1940) AATCCTGTATCAGCTGTGGT; Gphn reverse (2234-2253) TCATAGCCGTCCGATGACCA; Gapdh yielded an amplification product of 529 bp and Gphn of 332 bp.

Gel filtration of neuronal homogenates

Primary hippocampal neurons (1.2 x 10^6 cells / condition) pre-incubated with 50 µM 2-BP or the solvent overnight at DIV 14 were washed in cold PBS and lysed in 200 µl GeFi-buffer (25 mM Tris, pH 7.4, 100 mM NaCl, 5 mM EDTA) supplemented with 0.5 % Triton X-100, 0.5 % saponin and protease inhibitor cocktail (Roche, Mannheim, Germany). Cells were briefly sonicated and rotated for 1 h head-over-tail at 4 °C. Postnuclear fraction was loaded on a size-exclusion column (Superose 6 GL) connected to an ÄKTApurifier (GE Healthcare, Little Chalfont, UK) and run at 1 ml/min with GeFi-buffer. Fractions were collected in 600 µl steps, concentrated to 100 µl and equal volumes were resolved by SDS-PAGE. Elution times were calculated according to the

elution profile of standard proteins of 43, 75, 158, 440 and 669 kDa (Gel Filtration Calibration Kit, GE Healthcare).

Surface biotinylation assay

Two 6-well dishes with 500.000 neurons at 13 DIV each were used per condition. Neurons were pre-incubated with 50 µM 2-BP or the solvent overnight. All steps were performed at 4 °C and all solutions were precooled as low temperature is known to inhibit receptor membrane trafficking and endocytosis [2]. Neurons were rinsed twice with ice-cold PBS supplemented with 0.5 mM $MgCl_2$ and 1 mM $CaCl_2$. Surface proteins were biotinoylated in a 20 min incubation step at 4 °C in 1 ml PBS supplemented buffer containing 1mg/ml sulfo-NHS-SS-biotin (Thermo Scientific, Rockford, IL). Unreacted biotin reagent was quenched by washing with 100 mM glycine dissolved in supplemented PBS buffer. Cells were lysed in 500 µl PBS containing 2% Triton X-100, 0.2% SDS and protease inhibitor cocktail (Roche, Mannheim, Germany). After brief sonication, cells were incubated for 1 h on ice and subjected to centrifugation (10000 xg for 2 min). 50 µl were saved as loading control and supernatants were incubated with Neutravidin beads (Thermo Scientific, Rockford, IL) for 2 h at room temperature or overnight at 4 °C.

1. Fang C, Deng L, Keller CA, Fukata M, Fukata Y, et al. (2006) GODZ-mediated palmitoylation of GABA(A) receptors is required for normal assembly and function of GABAergic inhibitory synapses. J Neurosci 26: 12758-12768.

2. Kittler JT, Thomas P, Tretter V, Bogdanov YD, Haucke V, et al. (2004) Huntingtin-associated protein 1 regulates inhibitory synaptic transmission by modulating gamma-aminobutyric acid type A receptor membrane trafficking. Proc Natl Acad Sci U S A 101: 12736-12741.
